# Twenty six new species of Leioproctus (Colletellus): Australian Neopasiphaeinae, all but one with two submarginal cells (Hymenoptera, Colletidae, *Leioproctus*)

**DOI:** 10.3897/zookeys.811.28924

**Published:** 2018-12-31

**Authors:** Remko Leijs, James Dorey, Katja Hogendoorn

**Affiliations:** 1 South Australian Museum, North Terrace, Adelaide, SA 5000, Australia South Australian Museum Adelaide Australia; 2 School of Biology, Flinders University, Adelaide, SA 5001, Australia Flinders University Adelaide Australia; 3 School of Agriculture, Food and Wine, The University of Adelaide, SA 5005, Australia The University of Adelaide Adelaide Australia

**Keywords:** Bush Blitz surveys, colletid bees, identification keys, Leioproctus (Colletellus)

## Abstract

Twenty six new species of Australian Leioproctus (subgenusColletellus) (Hymenoptera, Colletidae) are described: *aberrans* Leijs, **sp. n.**, *alatus* Leijs, **sp. n.**, *albipilosus* Leijs, **sp. n.**, *albiscopis* Leijs, **sp. n.**, *aliceafontanus* Leijs, **sp. n.**, *altispinosus* Leijs, **sp. n.**, *aratus* Leijs, **sp. n.**, *auricorneus* Leijs, **sp. n.**, *bidentatus* Leijs, **sp. n.**, *centralis* Leijs, **sp. n.**, *ciliatus* Leijs, **sp. n.**, *claviger* Leijs, **sp. n.**, *consobrinus* Leijs, **sp. n.**, *constrictus* Leijs, **sp. n.**, *laciniosus* Leijs, **sp. n.**, *longivultu* Leijs, **sp. n.**, *lucidus* Leijs, **sp. n.**, *nitidifuscus* Leijs, **sp. n.**, *pectinatus* Leijs, **sp. n.**, *pilotapilus* Leijs, **sp. n.**, *quadripinnatus* Leijs, **sp. n.**, *rubicundus* Leijs, **sp. n.**, *rubricinctus* Leijs, **sp. n.**, *similis* Leijs, **sp. n.**, *splendens* Leijs, **sp. n.**, *submetallicus* Leijs, **sp. n.** High resolution images of diagnostic characters for all type specimens are included. Identification keys are provided.

## Introduction

The monotypic subgenus Leioproctus (Colletellus) was established by Michener (1965) for *L.velutinellus*, an unusual species of Australian neopasiphaeine bee with two submarginal cells and ciliate, rather than pectinate, inner hind tibial spurs. Formerly *Leioproctus* was classified under the Colletinae (Michener 2007), but recent phylogenetic analyses of the world’s Colletidae indicated its position under Neopasiphaeinae (Almeida et al. 2011). Now, more than 50 years later, examination of numerous specimens collected by T.F. Houston (WA-Museum) and others, as well as specimens collected on a number of Bush Blitz surveys in remote locations of Australia indicates that L. (Colletellus), is a rather speciose group of bees. The latter surveys are the result of a partnership between the Australian Government, BHP Billiton and Earthwatch Australia to document fauna and flora from selected national reserves. These surveys regularly result in the discovery of new invertebrate species (e.g., true bugs: Symonds and Cassis 2014; spiders: Baehr et al. 2013; bees: Hogendoorn et al. 2015, Leijs and Hogendoorn 2016; review of new described species: Taylor et al. 2017a, b).

Below, we describe 26 new species. Morphologically, the bees treated here key out to L. (Colletellus) when using Michener’s (2007) identification key to the subgenera of *Leioproctus* of the Australian Region, with the exception of a single character: not all females have a ciliate hind tibial spur. Removing the latter character, the distinctive characters for the subgenus are a combination of two submarginal cells, convex clypeus and supra clypeal area, a large, parallel-sided stigma and the jugal lobe of the hind wing long, i.e. extending well below the level of cu-v.

## Materials and methods

For descriptions of the new species the terminology used by Michener (2007) was followed. A Leica stereomicroscope with auto-montage imaging stacking software was used to obtain high-resolution images of all species. A compound microscope (Nikon, Eclipse 50i) and Zerene Stacker was used to image male genitalia and metasomal sterna seven and eight. Head measurements were taken from high-resolution frontal head images using the Leica auto-montage software. All measurements were converted relative to the head width, which was set to 50 units (following Houston 1990).

Abbreviations for these relative measurements are as follows:

**AOD** antennocular distance;

**ASD** antennal socket diameter;

**HL** head length;

**HW** head width;

**IAD** interantennal distance;

**LFW** lower face width, measured between lowest eye margins;

**OOD** ocellocular distance;

**OAD** ocellantennal distance;

**UFW** upper face width, measured between upper eye margins;

**OW** width of ocellar cluster.

Abbreviations for wing measurements are:

**MSR** ratio of stigma length and marginal cell length measured on wing costa;

**FSR** ratio of the lengths of the first submarginal cell and second submarginal cell;

**SFR** ratio of the lengths of the stigma and first submarginal cell.

Other abbreviations used are:

**T1, T2**, etc. first, second metasomal terga, etc.;

**S1, S2**, etc. first, second metasomal sterna, etc.;

**F1, F2**, etc. first, second flagellar segment, etc;

**BTP** basitibial plate.

**Figure 1.**
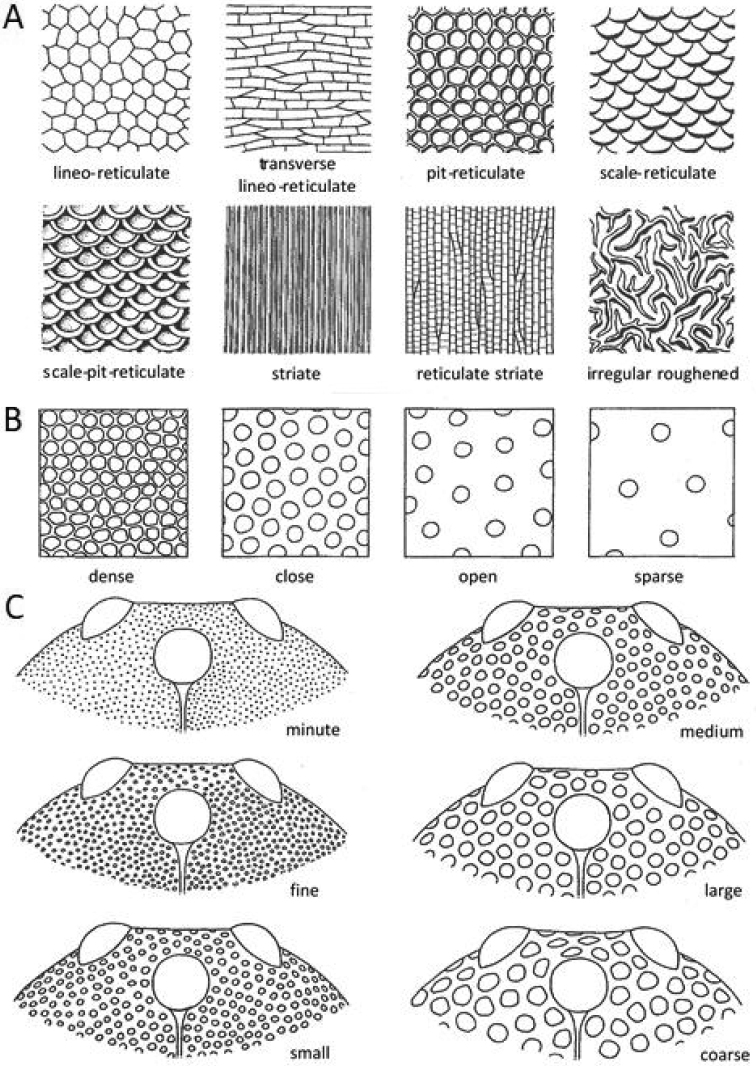
**A** Various kinds of integument sculpture **B** Grades of pit density **C** Grades of pit size. Reproduced from Houston (1975), with permission from CSIRO Publishing.

The terminology for integument sculpture, grades of pit and pubescence density and pit size follows Houston (1975; Fig. 1). Integument sculpture was observed using 40× magnification and YK-B144T LED ring elimination.

Some of the specimens treated here were also submitted to BOLD (Barcode of Life Database) for DNA barcoding using the cytochrome c oxidase subunit 1 gene. Specimen details, including DNA sequence, collecting dates and locality information can be accessed in BOLD under the project Australian Bee Survey, e.g., http://www.boldsystems.org/index.php/Public_RecordView?processid=AUSBS145-12. AUSBS-numbers are presented under material examined.

Repositories:

**AM** Australian Museum, Sydney

**ANIC** Australian National Insect Collection, Canberra

**SAMA** South Australian Museum, Adelaide

**QM** Queensland Museum, Brisbane

**WAM** Western Australian Museum, Perth

## Results and discussion

Twenty-six new species of *Leioproctus* are described. All specimens of these species key out to subgenus Colletellus when using Michener’s (2007: 148) identification key to the subgenera of *Leioproctus* of the Australian region. The type species of L. (Colletellus), *L.velutinellus* Michener, 1965 was unique amongst *Leioproctus* species due to its possession of a combination of two, rather than three, submarginal cells, convex clypeal and supraclypeal area, large parallel-sided stigma, long jugal lobe of the hind wing and ciliate rather than pectinate inner hind tibial spurs and long and pointed basitibial plates in females. The 26 additional species described here all conform to these characters with the exception of the hind tibial spurs in females and the shape of the basitibial plates. While 10 of the newly described species have ciliate spurs, 9 have pectinate spurs, and 16 of the additional species have short and rounded basitibial plates. Morphological characters as well as molecular data indicate that these species are almost certainly not a monophyletic group, but multivariate analyses of a large number of body size measurements as well as characters with discrete states did not result in clear clusters of species.

Although it may be possible to separate species groups based on wing venation, we hesitate to do so without the inclusion of independent molecular data for the majority of the species. Until now fresh tissue is only available from four of the L. (Colletellus) species, mainly from specimens collected at Bush Blitz surveys, and these have been DNA barcoded. Neighbour joining analyses using PAUP* (Swofford 2001) based on the DNA sequence data available from BOLD that include 22 other Australian *Leioproctus* species showed two independent groups of L. (Colletellus) species amongst other *Leioproctus*. Although this analysis is preliminary and only based on four L. (Colletellus) species it supports the idea that L. (Colletellus) as it stands now is paraphyletic. One of the groups, consisting of three South Australian species (*L.aberrans*, *L.laciniosus* and *L.rubicundus*) have pectinate inner hind tibial spurs in females and have a wing venation that differs from all other species described here. If corroborated, this would also indicate that a reduction of the number of submarginal cells has happened multiple times independently within *Leioproctus*.

### Wing vein reduction

**Figure 2.**
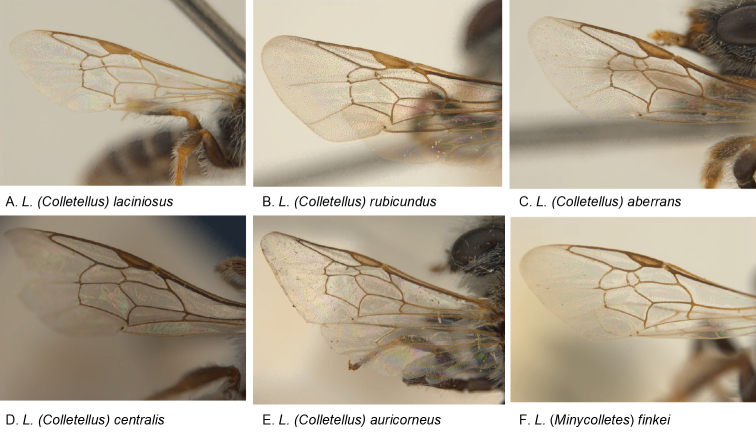
Variation in wing venation.

Wing vein reduction is a common phenomenon with reduction in body size in Hymenoptera (Danforth 1989). In L. (Colletellus) this seems to have occurred in two different ways. In the majority of the *Leioproctus* species with three submarginal cells, the first recurrent vein is distal to the first submarginal cross vein, as is also the case for most L. (Colletellus) species. It therefore seems that the majority of the L. (Colletellus) species lost the second submarginal cross vein (e.g., *L.centralis*: Fig. 2D). This is also demonstrated in species where the second submarginal cross vein is rudimentary (e.g., *L.auricorneus*: Fig. 2E). However, in the South Australian species *L.rubicundus* and *L.laciniosus* the first recurrent vein is basal to or meeting the submarginal cross vein (Fig. 2A, B), suggesting that in these species the first submarginal cross vein was lost. Regaining lost veins may be possible when considering the venation of *L.aberrans* (Fig. 2C), a species that has three submarginal cells and which is phylogenetically very close to *L.rubicundus* and *L.laciniosus*, uncorrected pairwise sequence divergence 6.0–8.3% (sequences available in BOLD). The same explanations are possible for other *Leioproctus* subgenera with two submarginal cells. In L. (Andrenopsis) and L. (Euryglossidia) the position of the first recurrent vein is distal to the first submarginal cross vein and it is therefore likely that these taxa lost the second submarginal cross vein, while L. (Baeocolletes) and L. (Filiglosssa) have possibly lost the first submarginal cross vein, because the position of the recurrent vein is basal or meeting the submarginal cross vein. Examination of Almeida’s Colletidae phylogeny suggest independent losses of submarginal cross veins for each of these above mentioned *Leiproctus* subgenera (Almeida et al. 2011).

### Distribution and phenology

**Figure 3.**
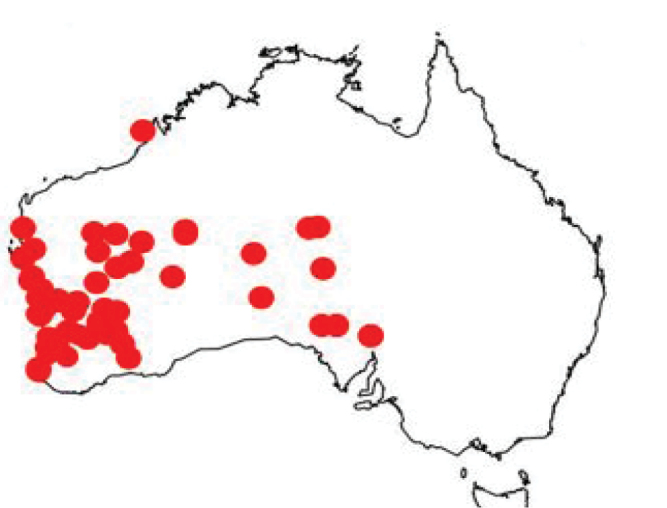
Distribution data of all Leioproctus (Colletellus) species combined.

**Figure 4.**
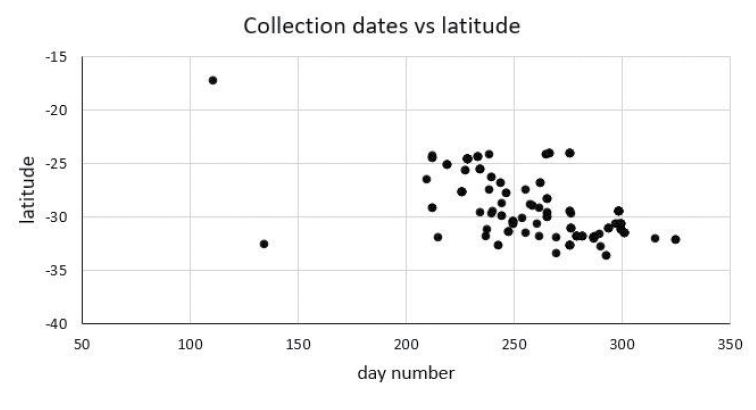
Leioproctus (Colletellus) collection dates plotted against the latitude of the collection sites.

Leioproctus (Colletellus) are widespread in arid and semi-arid environments of Western Australia, Northern Territory and South Australia west of approximately 138 degrees longitude (Fig. 3). The majority of the specimens were collected during the months August, September and October (Table 1), with a significant negative correlation between latitude and collection date (*r*^2^ = 0.4044; *p* < 0.001), Fig. 4), indicating a response in activity to temperature. The two outliers represent species *L.albipilosus* collected north of Broome in April, WA and a single male of *L.claviger* collected at Dawesville, WA in May. These records are at odds with most other records of these species, which show collecting dates between the end of July and late October.

**Table 1.** Phenology data of L. (Colletellus) in approximately 15-day periods per month.

Jan		Feb		Mar		Apr		May		Jun		Jul		Aug		Sep		Oct		Nov		Dec	
0	0	0	0	0	0	0	1	1	0	0	0	1	23	44	46	26	25	51	51	1	3	0	0

### Flower visitation

Leioproctus (Colletellus) were collected on 26 genera of plants belonging to 17 families (178 records). Eighty-five percent of the records involve only five plant families: Portulacaceae (24%), Proteaceae (22%), Stylidiaceae (17%), Asteraceae (13%) and Myrtaceae (8%). The Proteaceae and Myrtaceae species represent small shrubs, however the remaining 70 % of the records were from herbaceous plants, which seem to be preferred by L. (Colletellus) species. Five L. (Colletellus) species were collected on *Calandrinia* (Montiaceae).

Those species that have a multitude of flower records visited a multitude of plant families and genera, indicating that they are generalist flower visitors. However, lecty is often classified according to the number of plant genera and families from which pollen is collected (Cane and Sipes 2006) – information that is usually not recorded on the specimen labels. Therefore, we conclude that, currently, there is no evidence that L. (Colletellus) species are oligolectic.

## Systematics

### Revised identification key to the subgenera of *Leioproctus* for species with two submarginal cells (modified from Michener 2007)

N.B. Specimens of *L.aberrans* have three submarginal cells. While this species keys out to *Leioproctus* s. str. when using Maynard’s (2013) key to the subgenera of *Leioproctus* with three submarginal cells, it does not meet the other diagnostic characters for *Leioproctus* s. str., because the female facial fovea is absent, the antennal scape does not reach the median ocellus, apical hair bands are present on T2–4, and the male flagellum is short. Hence, the species is included here.

**Table:** 

1	Jugal lobe of hind wing extending well beyond level of cu-v	**L. (Colletellus)**
–	Jugal lobe of hind wing short, not attaining level of cu-v	**2**
–	Jugal lobe of hind wing meeting level of cu-v.	**4**
2	First recurrent vein basal to first submarginal cross vein (fig. 39–5h); clypeus and supraclypeal area usually flat, depressed, shining, largely impunctate; hind tibial spurs robust, curved apically, outer one nearly as coarsely toothed as inner	**L. (Baeocolletes)**
–	First recurrent vein distal to or meeting first submarginal cross vein; clypeus and supraclypeal area convex, the latter elevated above level of antennal sockets; hind tibial spurs not strongly curved apically, pectinate, outer one not coarsely toothed	**3**
3	Galea with several very long apical hairs and labial palpus filamentose, about as long as face	**L. (Filiglossa)**
–	Mouthparts not modified	**L. (Euryglossidia)**
4	Female: facial fovea broad, only slightly depressed. Male: S5 without apparent apical fringe, clypeus yellow	**L. (Andrenopsis)**
–	Female: facial fovea absent. Male: S5 with strong apical fringe, clypeus dark. Specimens of this species have been found with three submarginal cells	**L. (Minycolletes) abnormis**

#### Leioproctus Subgenus Colletellus

Taxon classificationAnimaliaHymenopteraColletidae

Michener, 1965

Leioproctus (Colletellus) Michener, 1965: 70.

##### Type species.

*Andrenopsisvelutinus* Cockerell, 1929 (not *Paracolletesvelutinus* Cockerell, 1929, homonym in *Leioproctus*) = *Leioproctusvelutinellus* Michener, 1965, by original designation.

**Diagnosis.** Two submarginal cells. Jugal lobe of hind wing extending well beyond level of cu-v. Antennal scape short, not reaching median ocellus. Facial fovea absent.

**Description.** The original description of L. (Colletellus) by Michener (1965), based on the type species *L.velutinellus* is no longer accurate with the addition of numerous species to this subgenus. Body length of 5–6.7 mm; eyes converging below; clypeus not protuberant; facial fovea absent; scape short, not approaching level of anterior ocellus, antenna as a whole short, median segments of flagellum much broader than long, with the exception of *L.aratus* which has an elongated flagellum with median segments longer than wide; two submarginal cells, however some species show incomplete or complete additional submarginal cross veins; stigma large, not parallel sided, more than half of length of costal edge of marginal cell; jugal lobe exceeding cu-v; metanotum not tuberculate; propodeum in profile with subhorizontal surface, curving onto vertical surface without sharp differentiation; inner hind tibial spur ciliate or pectinate; basitibial plate elongated and pointy or short and rounded; scopa plumose; claws cleft; metasoma in most species without distinct hair bands, but occasionally faint apical hair bands laterally, with the exception of *L.aberrans* and *L.laciniosus* which have distinct apical hair bands on T2–4; post gradular areas often depressed, especially in males, with orange-brown integumental colouring; large variation in the shape of male S7, especially with regard to the shape , the number of lateral lobes and the placement of setae.

The species in this subgenus are not difficult to identify because there are clear differences among species in integumental structure, especially on the clypeus, frons, scutum, metapostnotum and metasoma, as well as remarkable diversity of shapes of the male S7.

#### Identification keys

The following keys are based on our current knowledge of the species. Considering the low numbers of specimens and localities of several species, as well as the fact that for a number of species only a single sex is known, one should be aware, when using the keys, of the high likelihood of encountering undescribed sexes of species or even new species not treated in this paper.

##### Identification key to the females of Leioproctus (Colletellus) (20)

The number in brackets shows the number of species relevant to the character choices in each couplet.

**Table:** 

1	Basitibial plate pointed (Fig. 31F), (11 spp.)	**2**
–	Basitibial plate rounded (9 spp.)	**12**
2	Inner hind tibial spur pectinate, teeth longer than spur diameter (2 spp.)	**3**
–	Inner hind tibial spur ciliate, teeth shorter than spur diameter (9 spp.)	**4**
3	Basitibial plate short, circa 1/5 of the tibial length; propodeum smooth, shiny, almost wholly vertical; T1 smooth, openly punctate, T2–4 closely punctate with underlying transverse reticulation	***albipilosus* sp. n.**
–	Basitibial plate long, about 1/3 of the tibial length; metapostnotum triangular shaped, dull, pit-reticulate; T1–4 closely punctate	***longivultu* sp. n.**
4	Thorax and head with faint metallic shine	***submetallicus* sp. n.**
–	Thorax and head without metallic shine (8 spp.)	**5**
5	Scutum shiny and smooth, openly to closely punctate (5 spp.)	**6**
–	Scutum dull, densely punctate (3 spp.)	**10**
6	Ventral margin of clypeus with two tubercles	***bidentatus* n. sp**
–	Ventral margin of clypeus without tubercles (4 spp.)	**7**
7	T1 smooth with sparse, minute punctures	***splendens* sp. n.**
–	T1 sculpture with open, fine punctures or lineo-reticulate (3 spp.)	**8**
8	Ocellocular area irregularly roughened near ocellus and shiny towards eye; frons coarsely reticulate striate (Fig. 8C); antenna long, scape reaching ocellus (Fig. 8E)	***albiscopis* sp. n.**
–	Ocellocular area not as above; frons not striate; antenna short, scape not reaching ocellus (2 spp.)	**9**
9	Ocellocular area pit-reticulate, shallower and shiny towards eye; frons coarsely pit-reticulate (Fig. 16C)	***claviger* sp. n.**
–	Ocellocular area dull, with dense minute punctures; frons with dense small punctures (Fig. 17C)	***consobrinus* sp. n.**
10	Scutum, scutellum and metanotum with dense short light brown pubescence; clypeus somewhat shiny, openly punctate with minute depressions between punctures (Fig. 31E)	***velutinellus* Michener**
–	Scutum, scutellum and metanotum pubescence short to medium length, openly to closely spaced; clypeus dull, finely reticulate (Figs 15E, 28E) (2 spp.)	**11**
11	Scutum, scutellum and metanotum pubescence short and white; T1–4 predominantly orange (Fig. 16A)	***ciliatus* sp. n.**
–	Scutum, scutellum and metanotum pubescence of medium length, light brown; T1–4 dark brown with transparent posterior margins (Fig. 28A)	***similis* sp. n.**
12	Inner hind tibial spur ciliate, teeth shorter than spur diameter (2 spp.)	**13**
–	Inner hind tibial spur pectinate, with robust teeth (7 spp.)	**14**
13	Terga depressed anteriorly; T1 shiny and openly punctate; basitibial plate less than 1/5 of tibial length; inner hind tibial spur with less than 10 slender teeth	***constrictus* sp. n.**
–	Terga not depressed anteriorly, T1 shiny and very sparsely punctate; basitibial plate elongated and slightly pointed, more than 1/4 of tibial length; inner hind tibial spur with more than 10 tiny teeth	***nitidifuscus* sp. n.**
14	Scutum punctation near parapsidal lines sparse to open (4 spp.)	**15**
–	Scutum punctation close to dense (3 spp.)	**18**
15	T1 smooth and sparsely punctate; T2–4 transparent posterior margins wide, through which adpressed pubescence on anterior margin of following terga are visible as hair bands	***lucidus* sp. n.**
–	T1 dull and densely punctate; T2–4 no hair bands on anterior margins. (3 spp.)	**16**
16	T2–4 without dense adpressed hair bands on posterior margins, only semi erect hairs present; clypeus closely punctate	***rubicundus* sp. n.**
–	T2–4 with adpressed hair bands on posterior margins, T2 laterally only; clypeus openly punctate (2 spp.)	**17**
17	Scape black, shiny anteriorly (ventrally) almost without punctures; 2 submarginal cells	***laciniosus* sp. n.**
–	Scape dark brown, dull with microsculpture and punctures; 3 submarginal cells (Fig. 2C)	***aberrans* sp. n.**
18	Ocellocular area openly punctate (Fig. 23C); scutum pubescence medium length and open (Fig. 23A)	***pectinatus* sp. n.**
–	Ocellocular area impunctate, smooth or minutely reticulate (Figs 14C, 24C); scutum pubescence short (Figs 14A, 24A) (2 spp.)	**19**
19	Ocellocular area dull, finely pit-reticulate (Fig. 24C); terga brown-black with transparent orange posterior margins and open semi erect hair bands on T3–4	***pilotapilus* sp. n.**
–	Ocellocular area shiny, almost without punctures (Fig. 14C); terga predominantly orange with close semi erect hair bands on T3–4	***centralis* sp. n.**

##### Identification key to the males of Leioproctus (Colletellus) (17)

**Table:** 

1	Ocellocular area dull, finely to coarsely roughened (6 spp.)	**2**
–	Ocellocular area shiny, openly to sparsely punctate and/or with microsculpture (11 spp.)	**7**
2	Scutum dull, densely punctate or reticulate (4 spp.)	**3**
–	Scutum shiny, openly to closely punctate (2 spp.)	**5**
3	F1 longer than F2; clypeus punctation open to sparse; terga anteriorly with strongly depressed pregradular grooves, orange (Fig. 31G, H)	***velutinellus* Michener**
–	Length F1 equal to or shorter than F2; clypeus punctation close; terga anteriorly depressed (Figs 6B, 27B) or not depressed. (2 spp.)	**4**
4	Terga anteriorly depressed; posterior margin of terga transparent orange; head and scutum without faint metallic shine. (2 spp.)	**5**
–	Terga anteriorly not depressed; posterior margin of terga opaque brown; head and scutum with faint metallic shine	***submetallicus* sp. n.**
5	T1 shiny, posteriorly on disc with fine reticulation; supraclypeal area shiny; inner hind femur convex	***alatus* sp. n.**
–	T1 dull, posterior on disc with dense reticulation; supraclypeal area dull; inner hind femur concave	***rubricinctus* sp. n.**
6	Ocellocular area coarsely reticulate (Fig. 11C); metapostnotum smooth with shallow microsculpture (Fig. 11D)	***aratus* sp. n.**
–	Ocellocular area reticulate (Fig. 16I); metapostnotum dull and finely reticulate (Fig. 16J)	***claviger* sp. n.**
7	Metapostnotum dull, alveolate or coarsely reticulate (2 spp.)	**8**
–	Metapostnotum shiny, completely smooth or with microsculpture (9 spp.)	**9**
8	Basitibial plate pointed; metapostnotum regularly pit-reticulate; T2–3 dull with fine punctation and reticulation, pregradular areas strongly depressed	***bidentatus* sp. n.**
–	Basitibial plate rounded; metapostnotum irregularly coarsely reticulate; T2–3 shiny with close punctation, pregradular areas depressed	***altispinosus* sp. n.**
9	Ocellocular area smooth, almost without punctures (4 spp.)	**10**
–	Ocellocular area punctate or with microsculpture (5 spp.)	**13**
10	T1 openly punctate, T2–3 anteriorly strongly depressed	***constrictus* sp. n.**
–	T1 densely punctate or with microsculpture; T2–3 not anteriorly depressed (3 spp.)	**11**
11	T1 shiny, finely lineo-reticulate	***rubicundus* sp. n.**
–	T1 dull, densely punctate (2 spp.)	**12**
12	S2–4 apical hair bands with dense, short hairs, 2 submarginal cells (Fig. 2A)	***laciniosus* sp. n.**
–	S2–4 apical hair bands with longer, openly placed hairs, often 3 submarginal cells (Fig. 2C)	***aberrans* sp. n.**
13	Metapostnotum entirely smooth (2 spp.)	**14**
–	Metapostnotum with microsculpture (3 spp.)	**15**
14	Flagellum F1–5(6) orange; T1–2 dull, densely reticulate	***auricorneus* sp. n.**
–	Flagellum entirely dark; T1–2 smooth openly punctate	***quadripinnatus* sp. n.**
15	T2–4 anteriorly depressed, orange; ocellocular area smooth with open punctures	***centralis* sp. n.**
–	T2–4 anteriorly moderately depressed, same colour as disk; ocellocular area with microsculpture (2 spp.)	**16**
16	Propodeal triangle bordered by coarsely alveolate groove (Fig. 24J)	***pilotapilus* sp. n.**
–	Propodeal triangle bordered by fine alveolate groove (Fig. 9D)	***aliceafontanus* sp. n.**

### Leioproctus (Colletellus) aberrans

Taxon classificationAnimaliaHymenopteraColletidae

Leijs sp. n.

http://zoobank.org/7D5CDCA8-90E4-4EC8-A0D6-DD1086550DBA

Figures 2C , 5A–O

#### Specimens examined.

(1♂, 1♀): Female holotype: Bon Bon Stn (30.7789S; 135.3841E), 27 Oct. 2010, Leijs, R., on *Angianthusbrachypappus*, SAMA 32-033494, BOLD: AUSBS124-12/RL1629A.

Male allotype: Bon Bon Stn (30.7789S; 135.3841E), 27 Oct. 2010, Leijs, R., on *Angianthusbrachypappus*, SAMA 32-033495, BOLD: AUSBS125-12/RL1629B.

#### Diagnosis.

Three submarginal cells, distinct posterior hair bands on T2–4.

#### Description.

Holotype, female, body length: 6.2 mm; head width: 2 mm. *Relative head measurements*: HW 50, ASD 2.7, AOD 8.5, HL 36, IAD 9.4, LFW 29, OAD 15, OOD 9.7, OW 17, UFW 35, HW/HL 1.4, LFW/UFW 0.8. *Relative wing measurements*: MSR 1.56, FSR 1.76, SFR 0.83.

*Structure*: terga not depressed anteriorly; BTP rounded; BTP/tibial length ratio 0.2; inner hind tibial spur pectinate with 8 strong teeth.

*Sculpture*: scutum smooth with sparse punctures; metapostnotum smooth, shiny, horizontal part as long as vertical; T1 lineo-reticulate, T2–3 transverse lineo-reticulate; clypeus shiny, openly punctate, orange anterior rim; supraclypeal area shiny, closely punctate; labrum smooth orange-brown; ocellocular area smooth, shiny; frons smooth openly to sparsely punctate; scape shiny, almost no punctures.

*Coloration*: terga anterior brown-orange, posterior margins transparent white, T2–4 with white adpressed hair bands; scopa white; mandibles orange with brown tip; scape black, flagellum F1–3 black, F4–10 orange-brown below.

*Pubescence*: scutum: dispersed, short, branched; scutellum: dispersed, short, branched; metanotum: dispersed, short, branched; T2–4 with hair bands, long, branched.

#### Description.

Allotype male, body length: 5.2 mm; head width: 1.8 mm. *Relative head measurements*: HW 50, ASD 3.5, AOD 7.6, HL 38, IAD 9.4, LFW 27, OAD 14, OOD 9.4, OW 19, UFW 36, HW/HL 1.3, LFW/UFW 0.8. *Relative wing measurements*: MSR 1.61, FSR 1.76, SFR 0.83.

*Structure*: terga not depressed anteriorly; BTP rounded; flagellum F1–3 black, F4–10 brown below, shorter than wide; *S7*: dorsal apical lobe small, ventral apical lobe large, branched setae present on dorsal subcentral apical ridge and apical lobe of ventral apical lobe.

*Sculpture*: scutum smooth with sparse punctures; metapostnotum smooth, shiny, horizontal part little shorter than vertical; T1 lineo-reticulate, T2–3 transverse lineo-reticulate; clypeus shiny, openly punctate, anterior rim orange; supraclypeal area shiny, closely punctate; ocellocular area smooth, shiny, almost no punctures; frons smooth openly to sparsely punctate.

*Coloration*: terga anteriorly brown-orange; mandibles brown at base, orange medially with brown tip.

*Pubescence*: scutum: dispersed, short, branched; scutellum: dispersed, short, branched; metanotum: dispersed, short, branched; S5 with dense hair band, short, branched, S2–4 erect and open hair band; scape black, shiny, almost no punctures.

#### Remarks.

Both examined specimens have been DNA barcoded, accessible through the following links:

http://www.boldsystems.org/index.php/Public_RecordView?processid=AUSBS124-12

http://www.boldsystems.org/index.php/Public_RecordView?processid=AUSBS125-12

#### Flower records.

*Angianthusbrachypappus* (Asteraceae).

#### Distribution.

Figure 5O.

**Figure 5.**
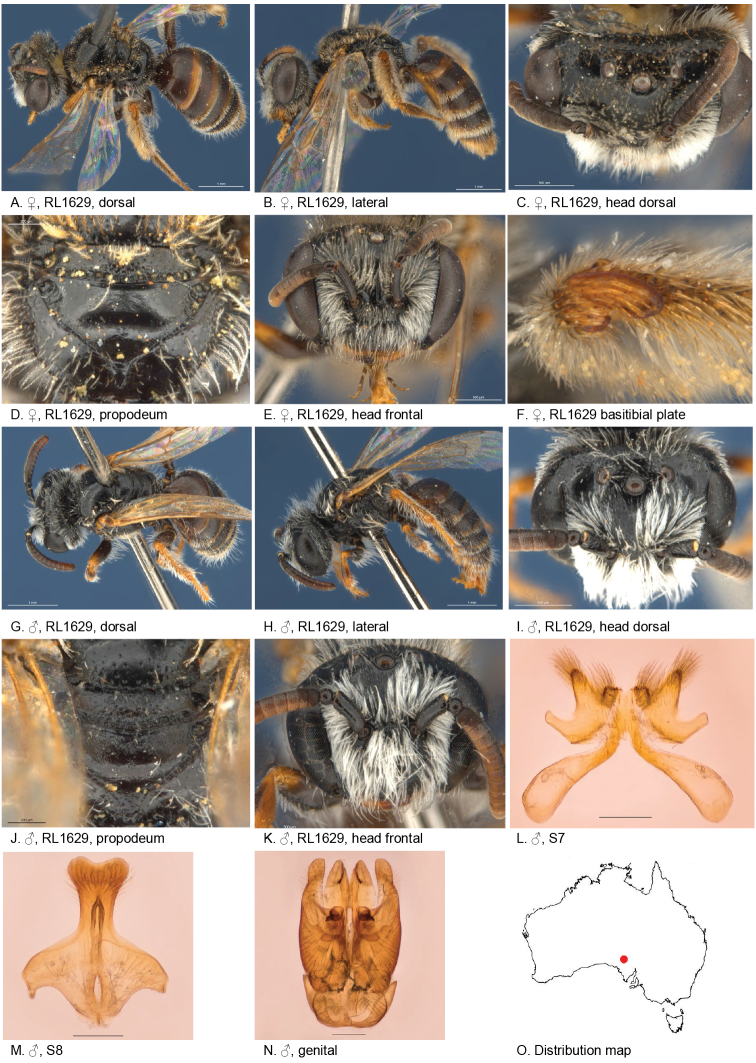
Leioproctus (Colletellus) aberrans Leijs, sp. n. ♀ holotype and ♂ allotype. Scale bar: 0.1 mm (**L, M, N**).

#### Etymology.

The specific epithet ‘*aberrans*’ refers to abnormal wing venation: this species has three submarginal cells instead of two (Fig. 2).

### Leioproctus (Colletellus) alatus

Taxon classificationAnimaliaHymenopteraColletidae

Leijs sp. n.

http://zoobank.org/51F9E4E1-F6A8-4AE0-8E52-FBD4A323316D

Figure 6A–I

#### Specimens examined.

(3♂): Holotype male, Kalbarri NP (27.8333S; 114.4667E), 14 Aug. 2003, Bickel, D., yellow pan trap, AM, K447299. Paratypes 2 males, same locality data as holotype, AM, K447298, K447306.

#### Diagnosis.

Head and thorax dull and densely reticulate, S7 with large wing shaped ventral apical lobes.

Female unknown.

#### Description.

male holotype: body length: 5.1 mm; head width: 1.5 mm. *Relative head measurements*: HW 50, ASD 4.0, AOD 6.9, HL 42, IAD 11.4, LFW 28, OAD 15, OOD 10.2, OW 19, UFW 38, HW/HL 1.2, LFW/UFW 0.7. *Relative wing measurements*: MSR 1.45, FSR 1.41, SFR 1.00.

*Structure*: terga anteriorly slightly depressed; BTP rounded, short; BTP/tibial length ratio 0.11; flagellum F1 = F2, other segments longer than wide; male S7: dorsal apical lobe absent, ventral apical lobe large, branched setae present on apico-medial area of ventral apical lobe.

*Sculpture*: scape dull, densely reticulate; scutum dull, anteriorly with transverse reticulation, densely punctate; metapostnotum pit-reticulate, some striae in lateral corners; T1 lineo-reticulate, T2–3 transverse lineo-reticulate; clypeus dull with dense reticulation; supraclypeal area dull with dense reticulation; ocellocular area concave, dull; frons densely sculptured.

*Coloration*: terga anterior dark brown, posterior margins transparent orange; mandibles and, F1–2 black, F3–11 orange-brown below.

*Pubescence*: scutum: open, short and scattered, long; scutellum: open, short and scattered, long; metanotum: open, short and scattered, long.

#### Flower records.

No data.

#### Distribution.

Figure 6I.

**Figure 6.**
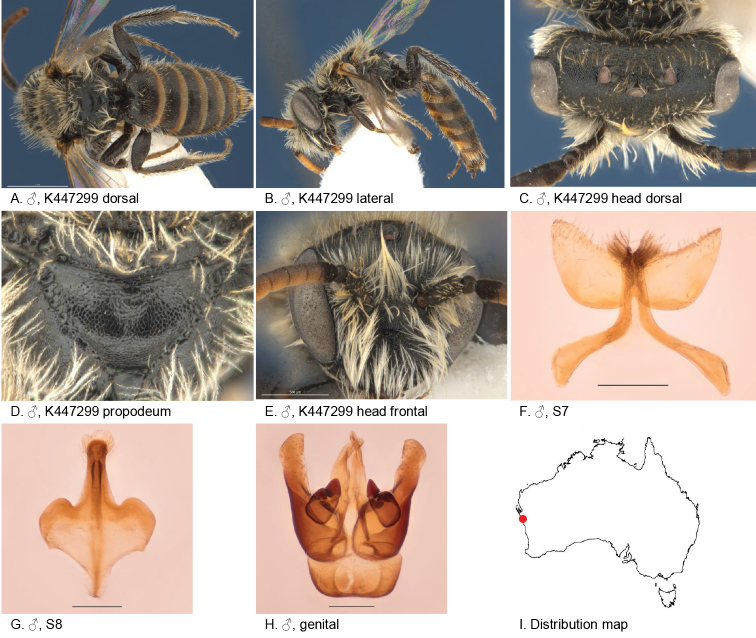
Leioproctus (Colletellus) alatus Leijs, sp. n. ♂ holotype. Scale bar: 0.1 mm (**F, G, H**).

#### Etymology.

The specific epithet refers to wing shaped lateral lobes of the male S7.

### Leioproctus (Colletellus) albipilosus

Taxon classificationAnimaliaHymenopteraColletidae

Leijs sp. n.

http://zoobank.org/751F411E-DBB3-46DF-9199-473971F65D74

Figure 7A–F

#### Specimens examined.

(1♀): Holotype female, 8 km S of Cape Bertholet (17.3167S; 122.1667E), WA, 21 Apr. 1977, Colless, D.H., ANIC 32-111660.

#### Diagnosis.

Short white pubescence on thorax and head, integument abdomen red, metapostnotum smooth and shiny.

Male unknown.

#### Description.

Female holotype: body length: 5 mm; head width: 1.7 mm. *Relative head measurements*: HW 50, ASD 3.2, AOD 7.9, HL 39, IAD 10.2, LFW 29, OAD 16, OOD 8.5, OW 19, UFW 33, HW/HL 1.3, LFW/UFW 0.9. *Relative wing measurements*: MSR 1.09, FSR 0.95, SFR 0.86.

*Structure*: terga anteriorly not depressed; BTP pointed, short; BTP/tibial length ratio 0.2; inner hind tibial spur pectinate with 7 strong teeth.

*Sculpture*: scutum smooth, closely punctate; metapostnotum smooth, shiny, almost wholly vertical; T1 lineo-reticulate, T2–3 transverse lineo-reticulate; clypeus smooth, closely punctate, ventral margin width 1/3 of ocellar diameter; supraclypeal area smooth, closely punctate; labrum dull, orange; ocellocular area smooth, openly to sparsely punctate; frons smooth closely punctate.

*Coloration*: terga anteriorly orange, posterior margins transparent orange; scopa white; mandibles orange with brown tip; scape brown with orange base; flagellum orange.

*Pubescence*: scutum: short, dense, white; scutellum: short, dense, white; S1–4 with fringes of long, branched hairs; scape, open, pubescence of white branched hairs.

#### Flower records.

No data.

#### Distribution.

Figure 7F.

**Figure 7.**
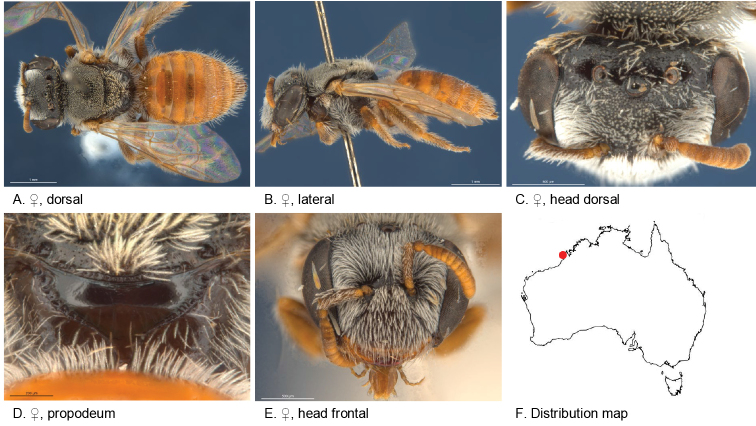
Leioproctus (Colletellus) albipilosus Leijs, sp. n. ♀ holotype ANIC 32-111660.

#### Etymology.

The specific epithet refers to short white pubescence on the scutum.

### Leioproctus (Colletellus) albiscopis

Taxon classificationAnimaliaHymenopteraColletidae

Leijs sp. n.

http://zoobank.org/B99A0ECC-6B1B-4665-9732-7B40A139A826

Figure 8A–F

#### Specimens examined.

(2♀): Holotype female, Arrowsmith River (29.6166S; 115.2881E), WA, 03 Oct. 1997, Houston, T.F., on *Allocasuarinacampestris*, WAM 19109; paratype female, same locality data as holotype, WAM 19108.

#### Diagnosis.

Integument mostly black, scopa white, ocellocular area shiny near eye, coarsely irregular roughened near ocellus; frons coarsely reticulate striate.

Male unknown.

#### Description.

Female holotype: body length: 6.2 mm; head width: 2 mm. *Relative head measurements*: HW 50, ASD 3.4, AOD 10.2, HL 36, IAD 8.4, LFW 32, OAD 12, OOD 8.8, OW 17, UFW 33, HW/HL 1.4, LFW/UFW 0.9. *Relative wing measurements*: MSR 1.88, FSR 1.00, SFR 1.00.

*Structure*: terga anteriorly not depressed; BTP pointed, broad; BTP/tibial length ratio 0.29; inner hind tibial spur ciliate; flagellum long, F4–9 slightly longer than wide; scape long, reaching ocellus, smooth.

*Sculpture*: scutum anteriorly transverse lineo-reticulate, remainder openly punctate; metapostnotum smooth, shallow lineo-reticulate; T1 lineo-reticulate, T2–3 transverse lineo-reticulate; clypeus shiny, with medium to large sparse punctures; supraclypeal area shiny, with medium to large sparse punctures; labrum brown-black; ocellocular area shiny near eye, coarsely irregular roughened near ocellus; frons coarsely reticulate striate.

*Coloration*: terga anteriorly brown black, posterior margins brownish, not transparent; scopa grey-white with darker pubescence towards BTP; mandibles brown.

*Pubescence*: scutum: medium length with sparse, long, grey brown hairs; scutellum: sparse, long, grey brown along edges; sterna white-grey fringe at S5.

#### Flower records.

*Allocasuarinacampestris* (Casuarinaceae).

#### Distribution.

Figure 8F.

**Figure 8.**
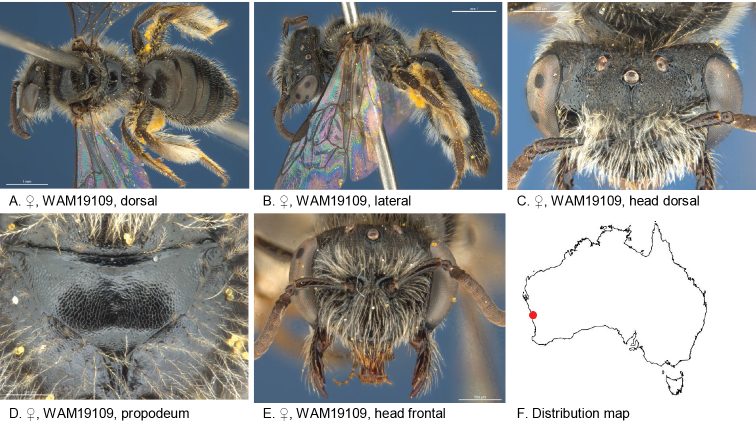
Leioproctus (Colletellus) albiscopis Leijs, sp. n. ♀ holotype.

#### Etymology.

The specific epithet refers to the white scopa pubescence on the hind tibia of the female.

### Leioproctus (Colletellus) aliceafontanus

Taxon classificationAnimaliaHymenopteraColletidae

Leijs sp. n.

http://zoobank.org/873313DC-5CD5-4FCE-8432-EFE2565E699C

Figure 9A–I

#### Specimens examined.

(7♂): Holotype male, James Ranges (24.25S; 133.43E), 22 Sep. 1978, Cardale, J., ANIC 32-111659;

Paratypes: 2 males, 56 km SE of Alice Springs (24.1833S; 134.0167E), 24 Sep. 1978, Cardale, J., ANIC 32-111667–68; 1 male, James Ranges (24.25S; 133.43E), 22 Sep. 1978, Cardale, J., ANIC 32-111669; 3 males, 45 km NE of Welbourn Hill (27.05S; 134.37E), 20 Sep. 1978, Cardale, J., ANIC 32-111670–72.

#### Diagnosis.

Ocellocular area smooth with some punctures near eye margin, metapostnotum shiny with microsculpture, posterior margins of terga transparent.

Female unknown.

#### Description.

Male holotype: body length: 5.1 mm; head width: 1.62 mm. *Relative head measurements*: HW 50, ASD 2.8, AOD 7.1, HL 38, IAD 9.2, LFW 27, OAD 13, OOD 10.5, OW 17, UFW 34, HW/HL 1.3, LFW/UFW 0.8. *Relative wing measurements*: MSR 1.15, FSR 0.96, SFR 1.00.

*Structure*: terga anteriorly moderately depressed; BTP rounded. S7: dorsal apical lobe absent, ventral apical lobe large, robust simple setae present on dorsal subcentral apical ridge and ventral apical lobe.

*Sculpture*: scutum smooth, with sparse fine punctures; metapostnotum dullish, finely reticulate; T1 lineo-reticulate, T2–3 transverse lineo-reticulate; clypeus smooth, openly to closely punctate; supraclypeal area smooth, openly punctate; labrum smooth; scape dull; ocellocular area shiny, minutely reticulate, with sparse punctures; frons smooth closely punctate.

*Coloration*: terga anteriorly dark brown, posterior margins transparent brown; mandibles brown with reddish tip; labrum brown; scape and flagellum brown.

*Pubescence*: scutum: medium length, open; scutellum: medium length, open.

#### Flower records.

No data.

#### Distribution.

Figure 9I.

**Figure 9.**
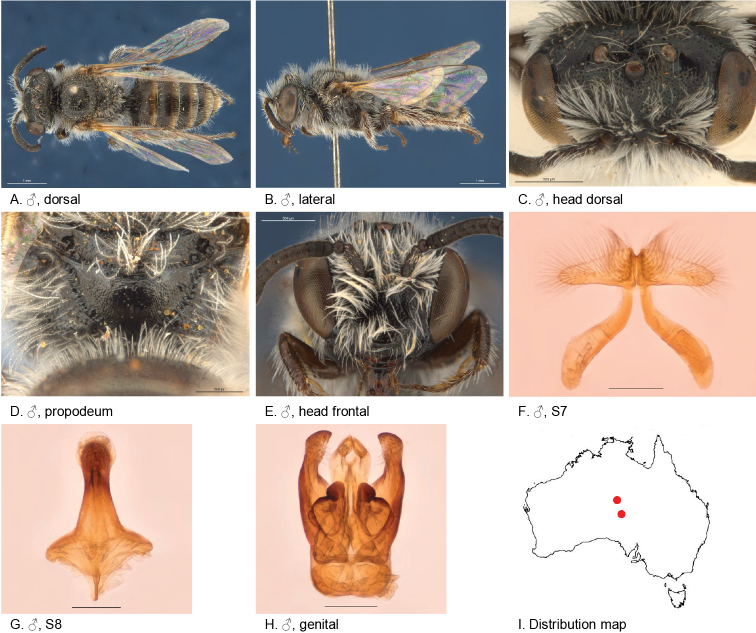
Leioproctus (Colletellus) aliceafontanus Leijs, sp. n. ♂ holotype ANIC 32-111659. Scale bar: 0.1 mm (**F,G,H**).

#### Etymology.

The specific epithet refers to the distribution of this species around Alice Springs.

### Leioproctus (Colletellus) altispinosus

Taxon classificationAnimaliaHymenopteraColletidae

Leijs sp. n.

http://zoobank.org/4E3CAA6B-279D-400E-B179-65167BBC06D4

Figure 10A–I

#### Specimens examined.

(28♂): Holotype male, 25 km SW of Tangadee (24.5688S; 118.7636E), 22 Aug. 1984, Houston, T.F. & Hanich, B.P., on *Dicrastylisflexuosa*, WAM 12368;

Paratypes: 24 males, 24 km NNE of Beyondie (24.7055S; 120.2558E), 17 Aug. 1984, Houston, T.F. & Hanich, B.P., on *Calandrinia*, WAM 12305–28; male, Tangadee (24.5688S; 118.7636E), 22 Aug. 1984, Houston, T.F. & Hanich, B.P., on *Calandrinia*, WAM 12330; male, 10 km NNW of Meedo (25.6991S; 114.7175E), 23 Aug. 1980, Howard, C.A. & Houston, T.F., on *Calandriniapolyandra*, WAM 19839; male, 5 km SSE of Eurardy HS (27.5472S; 114.6247E), 27 Aug. 1999, Houston, T.F., on *Calandrinia* , WAM 27540.

#### Diagnosis.

Ocellocular area shiny and openly punctate, metapostnotum coarsely reticulate, S7 lateral lobes very small relative to apodemes and with robust setae.

Female unknown.

#### Description.

Male holotype: body length: 5.8 mm; head width: 2 mm. *Relative head measurements*: HW 50, ASD 3.1, AOD 7.6, HL 40, IAD 8.7, LFW 28, OAD 15, OOD 8.8, OW 19, UFW 35, HW/HL 1.2, LFW/UFW 0.8. *Relative wing measurements*: MSR 1.43, FSR 0.97, SFR 0.89.

*Structure*: terga anteriorly depressed; BTP rounded; S7: dorsal apical lobe absent, ventral apical lobe small, very robust simple setae present on ventral apical lobe; scape roughened.

*Sculpture*: scutum closely punctate; metapostnotum dull, roughened; T1 lineo-reticulate, T2–3 transverse lineo-reticulate; clypeus shiny, closely punctate; supraclypeal area shiny, closely punctate; ocellocular area smooth openly punctate; frons densely punctate.

*Coloration*: terga anteriorly black, posterior margins orange, transparent; mandibles brown, orange tip; flagellum dark brown.

*Pubescence*: scutum: open, medium length, branched; scutellum: open, medium length, branched; metanotum: open, medium length, branched; sterna 5 with row of dense long straight hairs; scape with pubescence.

#### Remarks.

There is some variation in body size. There are a few exceptionally large males. Several specimens are carrying Strepsiptera.

#### Flower records.

*Calandriniapolyandra, Calandrinia* sp. (Montiaceae), *Dicrastylisflexuosa* (Lamiaceae).

#### Distribution.

Figure 10I.

**Figure 10.**
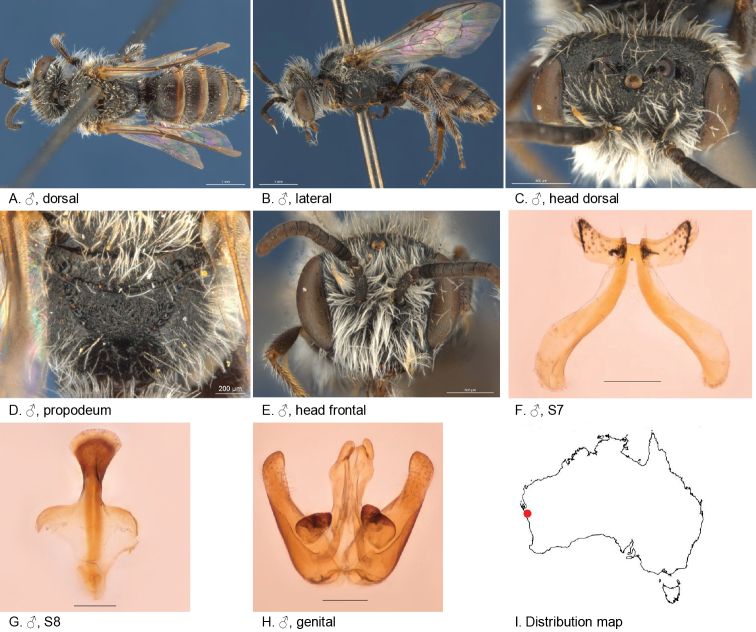
Leioproctus (Colletellus) altispinosus Leijs, sp. n. ♂ holotype WAM12368. Scale bar: 0.1 mm (**F, G, H**).

#### Etymology.

The specific epithet refers to the robust setae on the tip of the ventral apical lobe of male sterna 7. It also is a translation of the name of our linguistic advisor Wiebe Hogendoorn.

### Leioproctus (Colletellus) aratus

Taxon classificationAnimaliaHymenopteraColletidae

Leijs sp. n.

http://zoobank.org/71D12BE5-DFCD-4AA8-86F7-CA18F5F86FFB

Figure 11A–I

#### Specimens examined.

(1♂): Holotype male, Orange Grove (32.0233S; 116.0253E), 03 Aug. 1986, Peakall, R., on *Prasophyllumfimbria*, WAM 12365.

#### Diagnosis.

Antennae long, ocellocular area very roughly sculptured, metapostnotum smooth with shallow microsculpture, S7 with small and narrow dorsal and ventral lobes bearing robust simple setae.

Female unknown.

#### Description.

Male holotype: body length: 6 mm; head width: 1.85 mm. *Relative head measurements*: HW 50, ASD 3.1, AOD 6.1, HL 41, IAD 9.9, LFW 24, OAD 13, OOD 9.0, OW 17, UFW 34, HW/HL 1.2, LFW/UFW 0.7. *Relative wing measurements*: MSR 1.94, FSR 0.93, SFR 1.19.

*Structure*: terga anteriorly little depressed; BTP rounded, elongated; flagellum elongated: F3–11 little less than twice as long as wide, light-brown below; S7: dorsal apical lobe of medium size, ventral apical lobe slender, robust simple setae present on dorsal and ventral apical lobes.

*Sculpture*: scutum smooth, open, small punctures, posteriorly denser; metapostnotum smooth, shallow micro-sculpture; T1 lineo-reticulate, T2–3 transverse lineo-reticulate; clypeus smooth, openly to closely punctate; supraclypeal area smooth, openly punctate, medially without punctures; labrum black; ocellocular area coarsely pit-reticulate; frons coarsely pit-reticulate; scape closely punctate.

*Coloration*: terga anteriorly brown, posterior margins transparent orange; mandibles black with brown tip; scape black; F3–11 light-brown below.

*Pubescence*: scutum: medium length, open, brown; scutellum: medium length, open, brown.

#### Flower records.

*Prasophyllumfimbria* (Orchidaceae).

#### Distribution.

Figure 11I.

**Figure 11.**
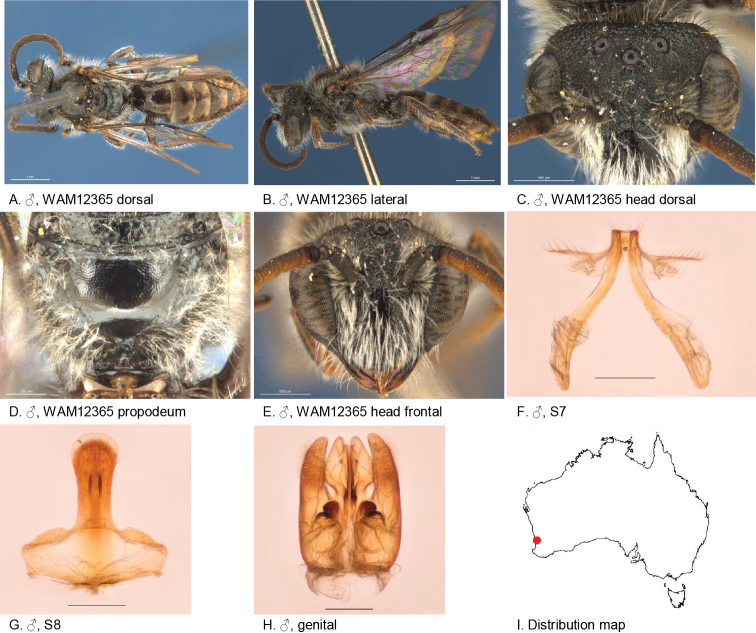
Leioproctus (Colletellus) aratus Leijs, sp. n. ♂ holotype. Scale bar: 0.1 mm (**F, G, H**).

#### Etymology.

The specific epithet refers to very coarse pit-reticulate sculpture on the head.

### Leioproctus (Colletellus) auricorneus

Taxon classificationAnimaliaHymenopteraColletidae

Leijs sp. n.

http://zoobank.org/58AC9DC5-F331-4D55-A84C-5225A8049ABA

Figures 2E , 12A–I

#### Specimens examined.

(1♂): Holotype male, 56 km SE of Alice Springs (24.1833S; 134.0167E), 03 Oct. 1978, Cardale, J., Malaise trap, ANIC 32-111658.

#### Diagnosis.

Ocellocular area smooth shiny openly punctate, metapostnotum shiny, but T1–2 dull and densely reticulate, flagellum orange. S7 ventral lobe large with long branched setae.

Female unknown.

#### Description.

Male holotype: body length: 4.9 mm; head width: 1.7 mm. *Relative head measurements*: HW 50, ASD 3.1, AOD 6.5, HL 40, IAD 10.8, LFW 26, OAD 16, OOD 9.2, OW 19, UFW 35, HW/HL 1.2, LFW/UFW 0.7. *Relative wing measurements*: MSR 1.22, FSR 0.96, SFR 0.96.

*Structure*: terga anteriorly almost not depressed; BTP rounded, elongated almost pointy; flagellum F1–7 orange, 8–11 brown; S7: dorsal apical lobe absent, ventral apical lobe large, branched setae present on posterior area of ventral apical lobe.

*Sculpture*: scutum smooth, with open small punctures; metapostnotum entirely smooth; T1 lineo-reticulate, T2–3 transverse lineo-reticulate; clypeus smooth, closely punctate; supraclypeal area smooth, closely punctate; ocellocular area smooth, openly punctate; frons smooth, openly to closely punctate.

*Coloration*: terga anteriorly black, posterior margins transparent brown; mandibles black with reddish tip; scape brown, dull.

*Pubescence*: scutum: long, open; scutellum: long, open; S5 posteriorly with very dense fringe of long white hairs.

#### Flower records.

No data.

#### Distribution.

Figure 12I.

**Figure 12.**
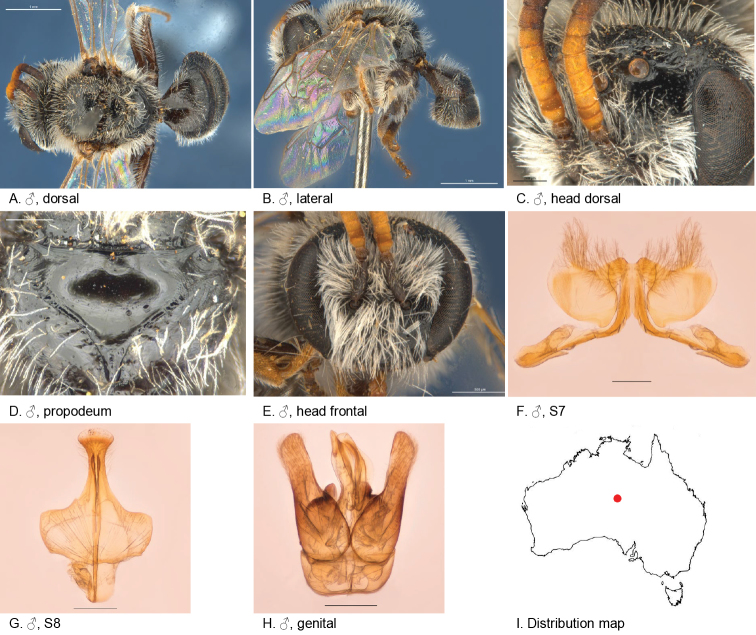
Leioproctus (Colletellus) auricorneus Leijs, sp. n. ♂ holotype ANIC 32-111658. Scale bar: 0.1 mm (**F, G, H**).

#### Etymology.

The specific epithet refers to the orange coloured antennae.

### Leioproctus (Colletellus) bidentatus

Taxon classificationAnimaliaHymenopteraColletidae

Leijs sp. n.

http://zoobank.org/9FE5BCA4-3E48-4DFB-A381-C4D035345763

Figure 13A–N

#### Specimens examined.

(8♂, 9♀): Holotype female, Gooseberry Hill (31.9541S; 116.0469E), 07 Oct. 1994, Houston, T.F., on *Verticordiaacerosa*, WAM 14412.

Allotype male, Cockleshell Gully (30.15S; 115.10E), 23 Sep. 1998, Houston, T.F., on *Stylidium*, WAM 20312.

Paratypes: 2 males, Cockleshell Gully (30.15S; 115.10E), 23 Sep. 1998, Houston, T.F., on *Stylidium*, WAM 20311, WAM 20313; male, Peak Charles National Park (32.8833S; 121.1608E), 18 Oct. 1985, Houston, T.F., on *Thryptomene*? , WAM 12400; male, Gooseberry Hill (31.9541S; 116.0469E), 07 Oct. 1994, Houston, T.F., on *Verticordiaacerosa*, WAM 14413; female, Arrowsmith River (29.6166S; 115.2881E), 03 Oct. 1997, Houston, T.F., on *Thysanotus*, WAM 19107; male, Mcdermid Rock (32.0222S 120.7339E), 27 Sep 1978, Houston, T.F., et al., *on Leptospermumerubescens*, WAM 19114; male, female, Lesmurdie (32.1300S; 116.0328E), 14 Oct. 2009, Batley, M., on *Stylidiumbulbiferum*, AM 359765, 359755; 2 females, Yallingup (33.6938S; 115.0358E), 20 Oct. 1983, Stoutamire, W.P., on *Agrostocrinum*, WAM 12381–2; 2 males, 3 females, Boorabbin Rock (31.2036S; 120.2856E), 04 Oct 1981, Houston, T.F., on *Baeckea*, WAM 12394–7, WAM 14421; male, Boorabbin Rock (31.2036S; 120.2856E), 04 Oct. 1981, Houston, T.F., on *Thryptomeneaustralis*, WAM 12398; female, Eneabba (29.8213S; 115.2692E), 04 Oct. 1985, McMillan, R.P., on *Thryptomene*, WAM 12399.

#### Diagnosis.

Clypeus of female with two small teeth at ventral margin, ocellocular area and clypeus smooth with open punctation, metapostnotum dull micro-alveolate. Male terga anteriorly strongly depressed, S7 ventral apical lobe large, branched setae present on dorsal subcentral apical ridge.

#### Description.

Female holotype: 6.6 mm; head width: 2.3 mm. *Relative head measurements*: HW 50, ASD 2.7, AOD 8.6, HL 35, IAD 10.5, LFW 29, OAD 14, OOD 10.6, OW 15, UFW 36, HW/HL 1.4, LFW/UFW 0.8. *Relative wing measurement*: MSR 1.26.

*Structure*: terga anteriorly little depressed; BTP pointed, long; BTP/tibial length ratio 0.33; inner hind tibial spur ciliate with circa 18 little teeth.

*Sculpture*: scutum smooth openly punctate; metapostnotum microalveolate, striate in lateral corners; T1 lineo-reticulate, T2–3 transverse lineo-reticulate; clypeus shiny, openly punctate, smooth between punctures, ventral margin drawn in thin plate with two teeth; supraclypeal area smooth, shiny; labrum smooth; ocellocular area smooth openly punctate; frons densely punctate; vertex roughened with punctures; scape dullish openly coarsely punctate.

*Coloration*: terga anteriorly dark, posterior margins transparent orange; scopa brown; mandibles black with brown tip; flagellum F1–3 black, F4–10 orange/brown below.

*Pubescence*: scutum: medium length, open; scutellum: medium length, open; metanotum: medium length, open; sterna 1–5 with fringes of long simple hairs, S5 not dense; scape with medium length grey pubescence.

#### Description.

Male allotype: body length: 5.8 mm; head width: 2.1 mm. *Relative head measurements*: HW 50, ASD 2.4, AOD 6.1, HL 36, IAD 10.2, LFW 26, OAD 15, OOD 11.9, OW 15, UFW 36, HW/HL 1.4, LFW/UFW 0.7. *Relative wing measurements*: MSR 1.26, FSR 1.08, SFR 0.69.

*Structure*: terga anteriorly strongly depressed; BTP pointed; flagellum F4–11 slightly longer than wide. S7: dorsal apical lobe absent, ventral apical lobe large, branched setae present on dorsal subcentral apical ridge; scape short.

*Sculpture*: scutum smooth openly punctate; metapostnotum dull, alveolate and striate in lateral corners; T1 lineo-reticulate, T2–3 transverse lineo-reticulate; clypeus shiny, openly punctate with smooth interspaces; supraclypeal area smooth, shiny; ocellocular area shiny, with open to sparse punctures; frons shiny, densely punctate; scape dull.

*Coloration*: terga anteriorly orange, posterior margins transparent orange; mandibles black with brown tip; flagellum black; scape black.

*Pubescence*: scutum: medium length, open; scutellum: medium length, open; metanotum: medium length, open; scape with long pubescence.

#### Flower records.

*Agrostocrinum* sp. (Hemerocallidaceae), *Baeckea* sp. (Myrtacea), *Leptospermumerubescens* (Myrtacea), *Stylidiumbulbiferum*, *Stylidium* sp. (Stylidiaceae), *Thryptomeneaustralis* (Myrtacea), *Thysanotus* sp. (Asparagaceae), *Verticordiaacerosa* (Myrtacea).

#### Distribution.

Figure 13J.

**Figure 13.**
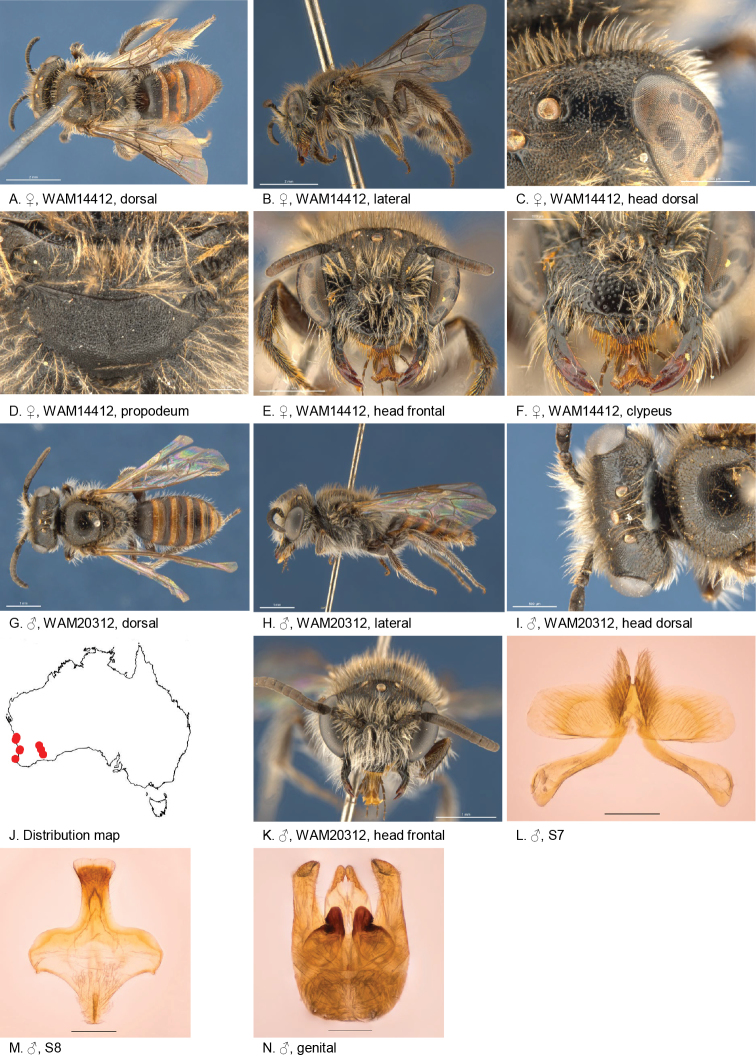
Leioproctus (Colletellus) bidentatus Leijs, sp. n. ♀ holotype and ♂ allotype. Scale bar: 0.1 mm (**L, M, N**).

#### Etymology.

The specific epithet refers to the two teeth on the ventral margin of the clypeus.

### Leioproctus (Colletellus) centralis

Taxon classificationAnimaliaHymenopteraColletidae

Leijs, n. sp.

http://zoobank.org/91E9360C-B1A4-4534-A9D4-3597C74BC5FA

Figures 2D , 14A–N

#### Specimens examined.

(5♂, 6♀): Holotype female, 56 km SE of Alice Springs (24.1833S; 134.0167E), 24 Sep. 1978, Cardale, J., ANIC 32-111661.

Allotype male, 37 km W of Glenayle HS (25.2666S; 122.0333E), 08 Aug. 1983, Houston, T.F. & McMillan, R.P., on *Calotismulticaulis*, WAM 12379;

Paratypes 3 females, 1 males, 56 km SE of Alice Springs (24.1833S; 134.0167E), 03 Oct. 1978, Cardale, J., Malaise trap, ANIC 32-111662,64–67; female, 56 km SE of Alice Springs (24.1833S; 134.0167E), 24 Sep. 1978, Cardale, J., on *Podolepiscanescens*, ANIC 32-111663; 3 males, 37 km W of Glenayle HS (25.2666S; 122.0333E), 08 Aug. 1983, Houston, T.F. & McMillan, R.P., on *Calotismulticaulis*, WAM 12375–78.

#### Diagnosis.

Ocellocular area smooth with a few dispersed punctures, metapostnotum dull fine transverse reticulate, terga with transparent posterior margins and depressed pregradular areas. Male S7 lateral lobes with very long branched setae.

#### Description.

Female holotype: body length: 6.5 mm; head width: 2.2 mm. *Relative head measurements*: HW 50, ASD 3.4, AOD 9.2, HL 39, IAD 9.1, LFW 30, OAD 16, OOD 10.0, OW 16, UFW 35, HW/HL 1.3, LFW/UFW 0.9. *Relative wing measurements*: MSR 1.14, FSR 0.97, SFR 0.86.

*Structure*: terga anteriorly little depressed; BTP rounded, elongated; BTP/tibial length ratio 0.26; inner hind tibial spur pectinate with 6 strong teeth.

*Sculpture*: scutum smooth, closely punctate; metapostnotum dullish, fine transverse reticulate; T1 lineo-reticulate, T2–3 transverse lineo-reticulate; clypeus smooth, openly to closely punctate; supraclypeal area smooth, openly to closely punctate; labrum dull, brown-orange; ocellocular area smooth without punctures; frons smooth closely punctate; scape brown, smooth and sparsely punctate ventrally.

*Coloration*: F3–10 orange below, anterior orange, posterior margins transparent orange, T3–4 white entire hair bands; scopa white; mandibles orange with black tip; scape brown.

*Pubescence*: scutum: short, close, orange-brown; scutellum: short, close, orange-brown; hair bands on T2 laterally; S1–4 with fringe of long straight branched hairs; scape dorsally with open pubescence of white branched hairs.

#### Description.

Male allotype: body length: 6.3 mm; head width: 1.95 mm. *Relative head measurements*: HW 50, ASD 2.7, AOD 8.3, HL 38, IAD 7.8, LFW 27, OAD 13, OOD 9.4, OW 18, UFW 35, HW/HL 1.3, LFW/UFW 0.8. *Relative wing measurements*: MSR 1.07, FSR 1.00, SFR 0.79.

*Structure*: terga anteriorly moderately depressed; BTP rounded; S7: dorsal apical lobe small, ventral apical lobe large, branched setae present on dorsal subcentral apical ridge and some on ventral apical lobe; scape brown, smooth and sparsely punctate.

*Sculpture*: scutum smooth, openly to closely punctate; metapostnotum dullish, with transverse irregular reticulation, rougher than female; T1 lineo-reticulate, T2–3 transverse lineo-reticulate; clypeus smooth, closely punctate; supraclypeal area smooth, closely punctate; ocellocular area smooth, sparsely punctate; frons smooth closely punctate.

*Coloration*: labrum orange; terga anteriorly brown-orange, posterior margins transparent orange; mandibles orange with brown tip; F4–11 orange brown below.

*Pubescence*: scutum: medium length, open, greyish brown; scutellum: medium, length open, greyish brown.

#### Flower records.

*Calotismulticaulis* (Asteraceae), *Podolepiscanescens* (Asteraceae).

#### Distribution.

Figure 14F.

**Figure 14.**
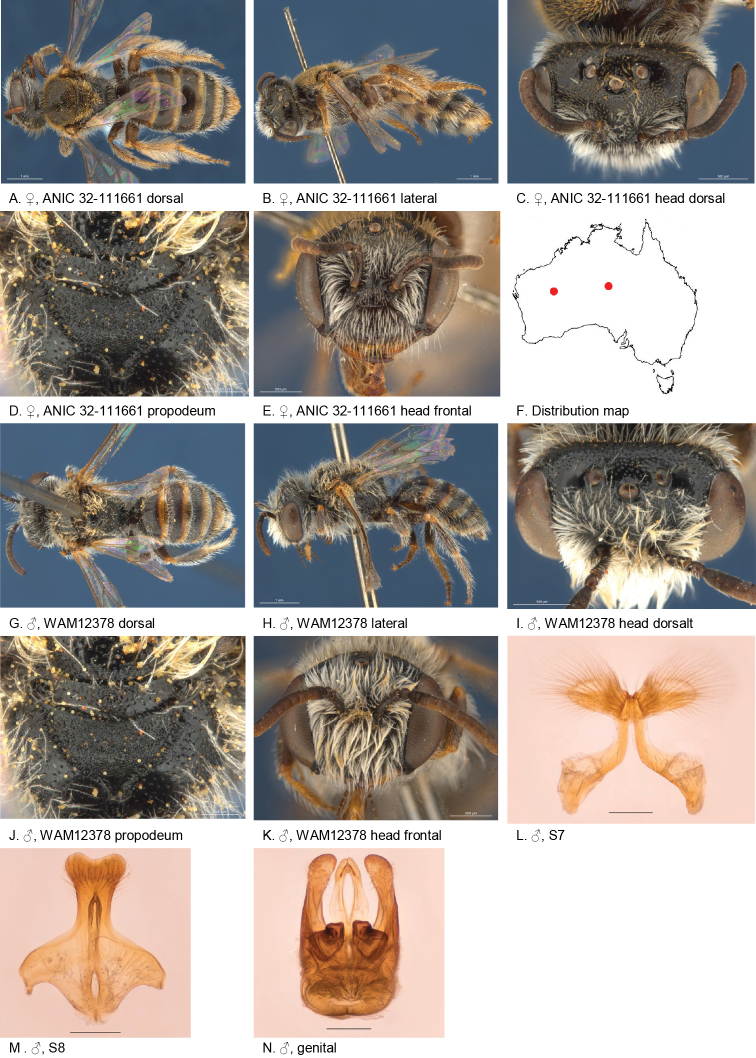
Leioproctus (Colletellus) centralis Leijs, sp. n. ♀ holotype and ♂ allotype. Scale bar: 0.1 mm (**L, M, N**).

#### Etymology.

The specific epithet refers to central Australian distribution of this species.

### Leioproctus (Colletellus) ciliatus

Taxon classificationAnimaliaHymenopteraColletidae

Leijs sp. n.

http://zoobank.org/8E7AAB7B-CBD7-491C-BC33-CD1AFD95FC8B

Figure 15A–F

#### Specimens examined.

(7♀): Holotype: female, East Yuna Nature Reserve (28.4191S; 115.2028E), 23 Sep. 1983, Houston, T.F. & C.A., on *Calandrinia*, WAM 12372;

Paratypes: 3 females, same locality data as holotype, WAM 12369–72; female, Kadji Kadji (29.1833S; 116.458E), 19 Sep. 2009, Leijs, R., blue pan trap, SAMA 32-033479; 2 females, Kadji Kadji (29.1392S; 116.3824E), 16 Sep. 2009, Leijs, R., on *Calandrinia* sp., SAMA 32-033480, BOLD: AUSBS284-13/ RL1503A, SAMA 32-033481 AUSBS285-13/ RL1503B.

#### Diagnosis.

Scutum with short grey pubescence, tergal integument reddish, ocellocular area dull, metapostnotum shiny and lineo-reticulate.

Male unknown.

#### Description.

Female holotype: body length: 5.2 mm; head width: 1.8 mm. *Relative head measurements*: HW 50, ASD 3.2, AOD 8.3, HL 39, IAD 9.4, LFW 31, OAD 14, OOD 8.9, OW 17, UFW 34, HW/HL 1.3, LFW/UFW 0.9. *Relative wing measurements*: MSR 1.43, FSR 1.13, SFR 0.96.

*Structure*: terga anteriorly not depressed; BTP pointed; BTP/tibial length ratio 0.31; inner hind tibial spur ciliate with circa 18 little teeth.

*Sculpture*: scutum dullish, densely punctate; metapostnotum dullish, lineo-reticulate; T1 lineo-reticulate, T2–3 transverse lineo-reticulate; clypeus dull, finely reticulate with sparse punctures; supraclypeal area dull, finely reticulate with sparse punctures; labrum smooth; ocellocular area dull, minutely reticulate; frons dull, pit-reticulate; scape dull minutely roughened..

*Coloration*: scape black; flagellum black, last 6 segments light brown below; labrum black; terga anterior brown-orange, posterior margins transparent orange; scopa light brown; mandibles black with brown tip.

*Pubescence*: scutum: short, close, white-grey; scutellum: short, close, white-grey.

#### Remarks.

Two specimens have been DNA barcoded, accessible through the following links:

http://www.boldsystems.org/index.php/Public_RecordView?processid=AUSBS284-13

http://www.boldsystems.org/index.php/Public_RecordView?processid=AUSBS285-13

#### Flower records.

*Calandrinia* sp. (Montiaceae).

#### Distribution.

Figure 15F.

**Figure 15.**
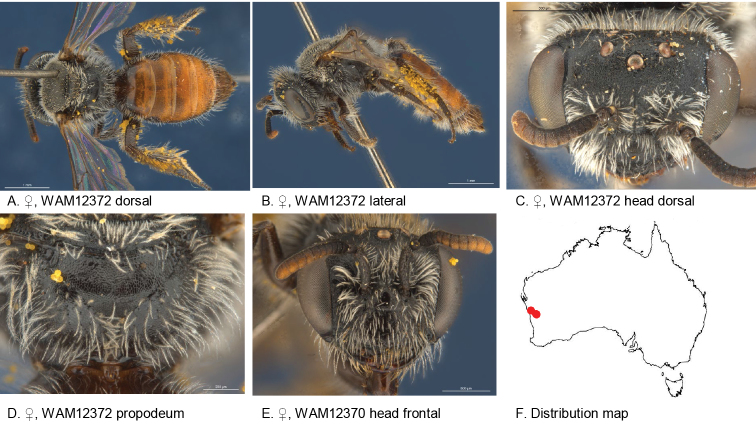
Leioproctus (Colletellus) ciliatus Leijs, sp. n. ♀ holotype.

#### Etymology.

The specific epithet refers to the ciliate inner hind tibial spur.

### Leioproctus (Colletellus) claviger

Taxon classificationAnimaliaHymenopteraColletidae

Leijs sp. n.

http://zoobank.org/43E63ECF-0947-4BBA-A296-F2B51569EB1A

Figure 16A–N

#### Specimens examined.

(6♂, 11♀): Holotype female, Dryandra (32.7802S; 116.9675E), 03 Oct. 1982, Howard, C.A. & Houston, T.F., on *Orthrosanthus*, WAM 12384;

Allotype male, Yanchep National Park (31.5350S; 115.6786E), 13 Sep. 1998, Houston, T.F., on *Stypandraglauca*, WAM 21486;

Paratypes: 7 females, Dryandra (32.7802S; 116.9675E), 03 Oct. 1982, Howard, C.A. & Houston, T.F., on *Orthrosanthus*, WAM 12383–90; 2 males, Dryandra (32.7802S; 116.9675E), Sep. 1977, McMillan, R.P., WAM 12391–2; male, Kings Park (31.9580S; 115.8331E), 25 Aug. 1998, Houston, T.F. et al., on *Thryptomenesaxicola*, WAM 18634; 2 females , Green Head (30.0650S; 114.9664E), 02 Sep. 1981, McMillan, R.P., WAM 18635, 19113; male, Dawesville (32.6494S; 115.6392E), 15 May 1980, Creagh, S., WAM 21487; female, Bindoo Hill (28.9161S; 115.1833E), 02 Sep. 1995, Cane, J. & Kervin, L., on *Keraudrenia*, WAM 32088; Durokoppin Nature Reserve (31.4102S; 117.7675E), 26 Aug. 1988, Hall, G.P., on *Baeckea*, WAM 12393.

#### Diagnosis.

Ocellocular area pit-reticulate, frons coarsely pit-reticulate, metapostnotum lineo-reticulate, female BTP pointy, hind tibial spur ciliate, male S7 with narrow club shaped ventral apical lobes.

#### Description.

Female holotype: body length: 6.3 mm; head width: 2.35 mm. *Relative head measurements*: HW 50, ASD 2.8, AOD 8.3, HL 37, IAD 11.0, LFW 30, OAD 12, OOD 10.5, OW 15, UFW 35, HW/HL 1.3, LFW/UFW 0.9. *Relative wing measurements*: MSR 1.60, FSR 1.23, SFR 0.95.

*Structure*: terga anteriorly little depressed; BTP pointed; BTP/tibial length ratio 0.29; inner hind tibial spur ciliate, circa 20 teeth.

*Sculpture*: scutum smooth, closely punctate; metapostnotum horizontal part pit-reticulate, vertical part transverse lineo-reticulate, lateral parts lineo-reticulate; T1 lineo-reticulate, T2–3 transverse lineo-reticulate; clypeus smooth with shallow microsculpture, openly punctate, ventral margin drawn into plate as wide as diameter of ocelli; supraclypeal area smooth with shallow microsculpture, medially without punctures; labrum smooth, black; ocellocular area pit -reticulate, shallower and more shine towards eye; frons pit-reticulate; scape closely punctate.

*Coloration*: terga anteriorly black-brown, posterior margins narrow, transparent orange; scopa brown; labrum black mandibles; black with brown tip; scape black; flagellum black.

*Pubescence*: scutum: very short, open and sparse, long, brown; scutellum: very short, open and sparse, long, brown.

#### Description.

Male allotype: body length: 5.8 mm; head width: 1.95 mm. *Relative head measurements*: HW 50, ASD 3.0, AOD 6.0, HL 40, IAD 11.9, LFW 27, OAD 13, OOD 10.9, OW 17, UFW 37, HW/HL 1.2, LFW/UFW 0.7. *Relative wing measurements*: MSR 1.67, FSR 1.10, SFR 1.03.

*Structure*: terga anteriorly depressed; BTP pointed; flagellum long, F3–11 longer than wide. S7: dorsal apical lobe absent, ventral apical lobe very narrow, setae absent; scape short.

*Sculpture*: scutum smooth, irregularly closely punctate; metapostnotum horizontal part pit-reticulate, vertical part transverse lineo-reticulate, lateral parts lineo-reticulate; T1 lineo-reticulate, T2–3 transverse lineo-reticulate; clypeus dull, large close punctures; supraclypeal area dull, irregular roughend; ocellocular area pit -reticulate, shallower and more shine towards eye; frons pit-reticulate; scape closely punctate.

*Coloration*: terga anteriorly black-brown, posterior margins transparent orange; mandibles black with brown tip; flagellum black.

*Pubescence*: scutum: medium length, open and sparse, light brown; scutellum: medium length, open and sparse, light brown; scape with long pubescence.

#### Flower records.

*Baeckea* sp. (Myrtacea), *Keraudrenia* sp. (Malvaceae), *Orthrosanthus* sp. (Iridaceae), *Stypandraglauca* (Hemerocallidaceae), *Thryptomenesaxicola* (Myrtacea).

#### Distribution.

Figure 16F.

**Figure 16.**
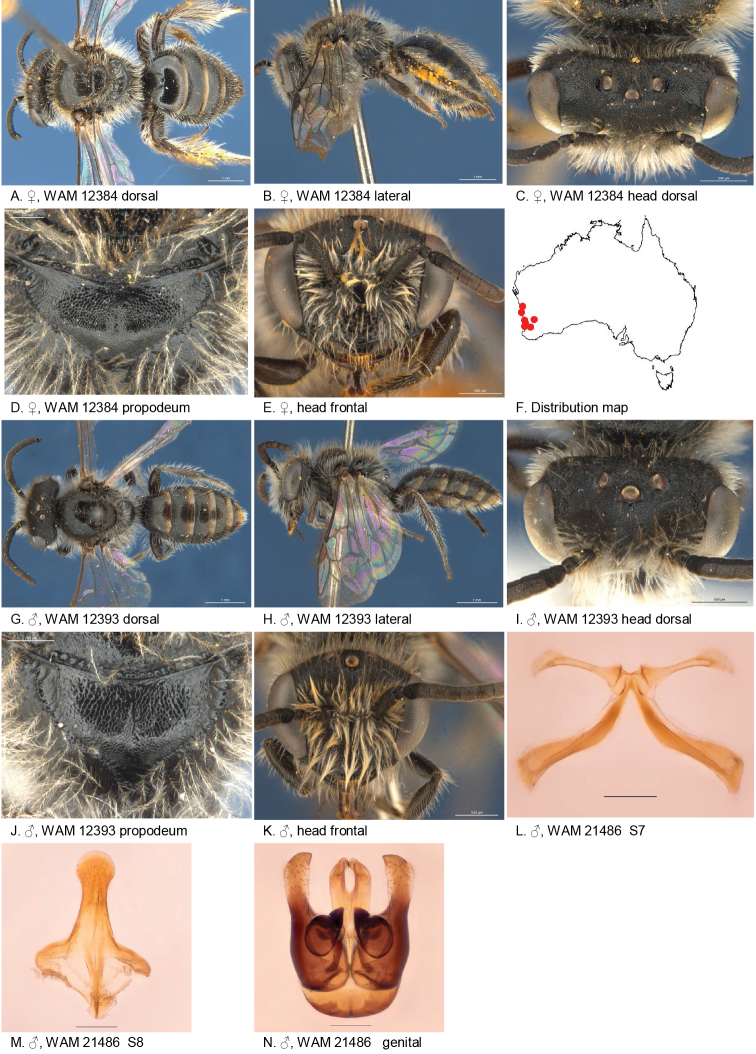
Leioproctus (Colletellus) claviger Leijs, sp. n. ♀ holotype and ♂ allotype and partaype. Scale bar: 0.1 mm (**L, M, N**).

#### Etymology.

The specific epithet refers to the club-shaped apical lobe of the male seventh sterna.

### Leioproctus (Colletellus) consobrinus

Taxon classificationAnimaliaHymenopteraColletidae

Leijs sp. n.

http://zoobank.org/EA941251-C89E-4173-AF0A-366B5BDC1E31

Figure 17A–F

#### Specimens examined.

(3♀): Holotype female, 115 km N of Wiluna (26.5961S; 121.3806E), 29 July 1983, Houston, T.F. & McMillan, R.P., on *Schoeniacassiniana*, WAM 19110; Paratypes: 2 females, 26 mi WNW of Wiluna (26.9438S; 120.3847E), 01 Sep. 1971, Houston, T.F., on *Pimelea*, WAM 19111–2.

#### Diagnosis.

Ocellocular area dull with minute dense punctures, BTP pointed, inner hind tibial spur ciliate, terga dark with narrow transparent posterior margins.

Male unknown.

#### Description.

Female holotype: body length: 6 mm; head width: 2 mm. *Relative head measurements*: HW 50, ASD 2.8, AOD 8.1, HL 37, IAD 10.9, LFW 32, OAD 12, OOD 9.5, OW 17, UFW 34, HW/HL 1.4, LFW/UFW 0.9. *Relative wing measurements*: MSR 1.84, FSR 1.03, SFR 1.13.

*Structure*: Terga anteriorly little depressed; BTP pointed; BTP/tibial length ratio 0.28; inner hind tibial spur ciliate with circa 18 small teeth.

*Sculpture*: scutum smooth, openly to closely punctate; metapostnotum lineo-reticulate; T1 lineo-reticulate, T2–3 transverse lineo-reticulate; clypeus smooth, openly to closely punctate, ventral margin broad; supraclypeal area smooth, sparsely punctate laterally; ocellocular area dull, dense minute punctures; frons densely small punctate; scape dull.

*Coloration*: terga anteriorly black-brown, posterior margins narrow, transparent orange; scopa light brown; labrum black; scape brown-black; flagellum black, apical 7 segments dark brown; mandibles black, medially brown, with black tip.

*Pubescence*: scutum very short, open and sparse, long, brown; scutellum very short, open and sparse, long, brown.

#### Flower records.

*Pimelea* sp. (Thymelaeaceae), *Schoeniacassiniana* (Asteraceae).

#### Distribution.

Figure 17F.

**Figure 17.**
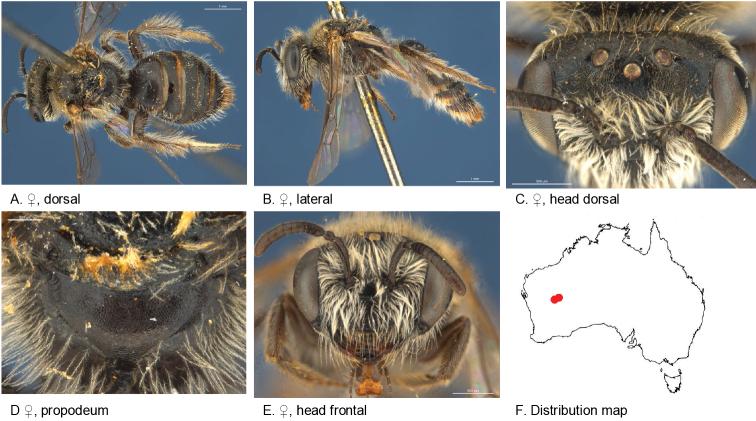
Leioproctus (Colletellus) consobrinus Leijs, sp. n. ♀ holotype WAM19110.

#### Etymology.

The specific epithet refers to the average L. (Colletellus) habitus of this species.

### Leioproctus (Colletellus) constrictus

Taxon classificationAnimaliaHymenopteraColletidae

Leijs sp. n.

http://zoobank.org/BFC7FD24-06E5-45DB-BB15-552C8AE08AEA

Figure 18A–M

#### Specimens examined.

(12♂, 29♀): Holotype female, 16 km WNW of Merredin (31.6169S; 118.3486E), 29 Oct. 1978, Houston, T.F., on *Grevilleaparadoxa*, WAM 12343;

Allotype male, WAM 12345, same locality data as holotype;

Paratypes: 6 females, 3 males, 16 km WNW of Merredin (31.6169S; 118.3486E), 29 Oct. 1978, Houston, T.F., on *Grevilleaparadoxa*, WAM 12339–42,44,46–49; 5 females, 3.5 km W of Yellowdine (31.3286S; 119.6503E), 27 Oct. 1978, Houston, T.F., on *Grevilleaparadoxa*, WAM 12350–4; female, 18 km SSW of Mulline (29.8686S; 120.3506E), 23 Sep. 1982, Houston, T.F. & Hanich, B.P., on *Eremophilapantoni*, WAM 12355; male, 1 km S of Evanston (29.7477S; 119.4814E), 23 Sep. 1982, Houston, T.F. & Hanich, B.P., on *Grevilleaparadoxa*, WAM 19116; 2 females, 3 males, Dowerin (31.1925S; 117.0367E), 21 Oct. 1984, McMillan, R.P., on *Grevillea*, WAM 19828–32; 14 females, 3 males, 29 km NE of Eneabba (29.5911S; 115.4397E), 26 Oct. 2000, Houston, T.F. & Mueller, O., on *Grevilleapetrophiloides*, WAM 31884-900; 1 male, Lochada, Omega track 1.5 km N of Mungada Rd, WA, (29.18332S; 116.45798E), 18 Sep. 2009, R. Leijs, on *Ecdeiocoleamonostachya*. SAMA 32-033499.

#### Diagnosis.

Ocellocular area, clypeus, scutum, metapostnotum and terga shiny with open to sparse punctures, post-gradular areas depressed, female BTP rounded, inner hind tibial spur ciliate, male S7 ventral apical lobes with long branched setae.

#### Description.

Female holotype: body length: 5.9 mm; head width: 1.9 mm. *Relative head measurements*: HW 50, ASD 2.6, AOD 7.9, HL 38, IAD 9.4, LFW 28, OAD 16, OOD 8.4, OW 17, UFW 32, HW/HL 1.3, LFW/UFW 0.9. *Relative wing measurements*: MSR 1.52, FSR 1.08, SFR 0.93.

*Structure*: terga anteriorly strongly depressed; BTP rounded; BTP/tibial length ratio 0.18; inner hind tibial spur ciliate with 5–10 slender teeth.

*Sculpture*: scutum smooth with sparse punctures; metapostnotum smooth, shiny, shallow pit-reticulate; T1 lineo-reticulate, T2–3 transverse lineo-reticulate; clypeus smooth with open to sparse punctures; supraclypeal area smooth, sparsely punctate; ocellocular area smooth, shiny, sparsely punctate; frons smooth openly punctate; vertex roughened with punctures.

*Coloration*: terga anteriorly brown-black, posterior margins transparent orange; scopa light brown; labrum brown; mandibles brown with darker tip; flagellum F9–11 orange/brown below.

*Pubescence*: scutum: dispersed, medium length, branched; scutellum: long, sparse, light brown; metanotum: medium length, branched, dense on posterior margin; sterna 1–4 with fringes of long simple hairs, S5 with fringe of dense branched white hairs; scape smooth, few punctures and a few short hairs.

#### Description.

Male allotype: body length: 4.2 mm; head width: 1.52 mm. *Relative head measurements*: HW 50, ASD 3.4, AOD 6.6, HL 38, IAD 10.3, LFW 26, OAD 16, OOD 8.9, OW 18, UFW 33, HW/HL 1.3, LFW/UFW 0.8. *Relative wing measurements*: MSR 1.61, FSR 1.00, SFR 1.00.

*Structure*: terga anteriorly strongly depressed; BTP rounded, slightly elongated. S7: dorsal apical lobe absent, ventral apical lobe medium large, branched setae present on entire ventral apical lobe.

*Sculpture*: scutum smooth, sparsely punctate; metapostnotum smooth with shallow microsculpture; T1 lineo-reticulate, T2–3 transverse lineo-reticulate; clypeus smooth, openly to closely punctate , ventral margin width about 1/3 ocellar diameter; supraclypeal area smooth with open to sparse punctures; ocellocular area smooth, almost no punctures; frons smooth openly to closely punctate; scape minutely roughened.

*Coloration*: terga anteriorly brown, posterior margins transparent orange; labrum black; mandibles brown with red-brown tip; flagellum brown, lighter towards the tip; scape black.

*Pubescence*: scutum: long sparse whitish; scutellum: long sparse whitish; metanotum medium length branched dense on posterior margin.

#### Flower records.

*Eremophilapantone* (Scrophulariaceae), *Grevilleaparadoxa* (Proteaceae), *Grevilleapetrophiloides* (Proteaceae), *Grevillea* sp. (Proteaceae), *Ecdeiocoleamonostachya* (Ecdeiocoleaceae).

#### Distribution.

Figure 18F.

**Figure 18.**
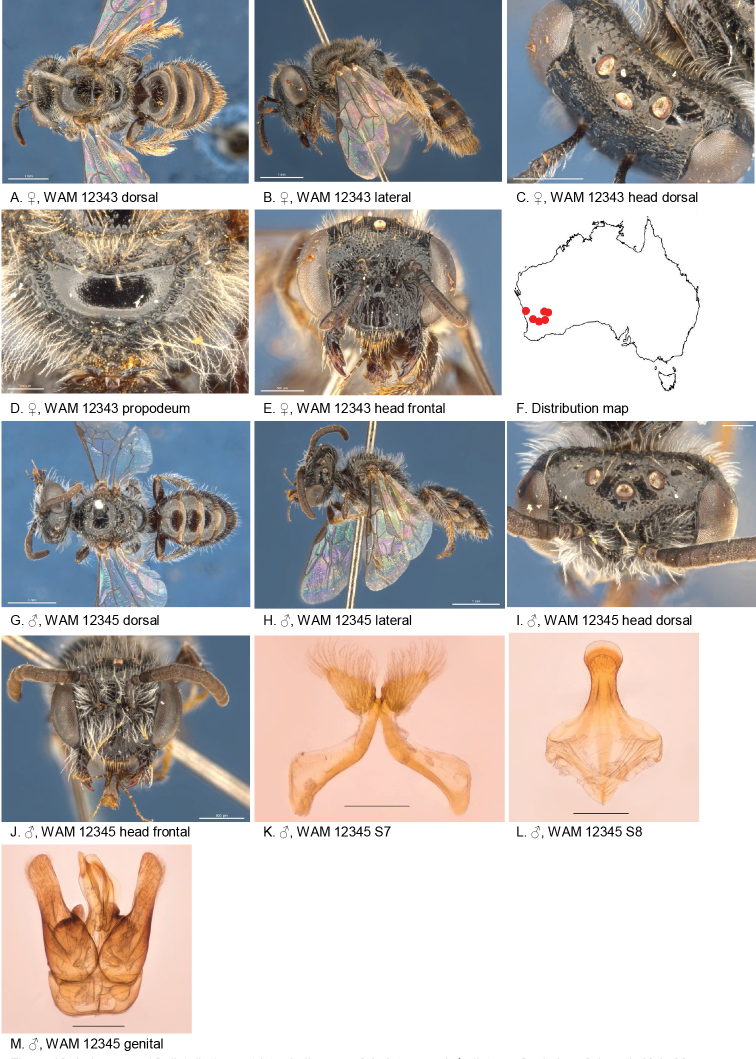
Leioproctus (Colletellus) constrictus Leijs, sp. n. ♀ holotype and ♂ allotype. Scale bar: 0.1 mm (**K, L, M**).

#### Etymology.

The specific epithet refers to strongly depressed post gradular areas of T2–3 in both sexes.

### Leioproctus (Colletellus) laciniosus

Taxon classificationAnimaliaHymenopteraColletidae

Leijs sp. n.

http://zoobank.org/6F55542F-8CED-4F4F-8C58-12E37AA81079

Figures 2B , 19A–M

#### Specimens examined.

(5♂, 3♀): Holotype female, Bon Bon Stn (30.7789S; 135.3841E), 26 Oct. 2010, Leijs, R., on *Angianthusbrachypappus*, SAMA 32-033484, BOLD: AUSBS140-12/RL1673A.

Allotype male, same locality data as holotype, SAMA 32-033486; BOLD: AUSBS143-12/RL1673B.

Paratypes: female, SAMA 32-033483, BOLD: AUSBS141-12/RL1673C, male, SAMA 32-033485, BOLD: AUSBS142-12/RL1673D, same locality data as holotype; female, Lake Wilson, 11 Sept 2015, SA, 26.0285S; 129.6159E, P. Hudson; 3 males, Ooldea (30.8915S; 132.0925E), 03 Oct. 1968, Key, Upton, Balderson, ANIC 32-111874–6;

#### Diagnosis.

First recurrent vein meeting to the first submarginal cross vein, T2–4 with adpressed hair bands on posterior margins, female BTB rounded, inner hind tibial spur pectinate.

#### Description.

Female holotype: body length: 5.6 mm; head width: 1.9 mm. *Relative head measurements*: HW 50, ASD 2.6, AOD 8.3, HL 35, IAD 9.4, LFW 28, OAD 15, OOD 9.1, OW 17, UFW 33, HW/HL 1.4, LFW/UFW 0.8. *Relative wing measurements*: MSR 1.39, FSR 1.71, SFR 0.79.

*Structure*: terga anteriorly not depressed; BTP rounded; BTP/tibial length ratio 0.22; inner hind tibial spur pectinate with 10 teeth.

*Sculpture*: scutum smooth with sparse punctures; metapostnotum smooth, shiny, horizontal part shorter than vertical; T1 lineo-reticulate, T2–3 transverse lineo-reticulate; scape dull with microsculpture; clypeus shiny, openly punctate; supraclypeal area shiny, some punctures; labrum smooth; ocellocular area smooth, shiny; frons smooth openly punctate.

*Coloration*: terga anteriorly brown-orange, posterior margins transparent white, T2–4 with white adpressed hair bands; scopa white; labrum orange clypeus with orange anterior rim; mandibles orange with black tip; scape brown; flagellum F1–3 black, F4–10 orange/brown below.

*Pubescence*: scutum: dispersed, medium length, branched; scutellum: dispersed, medium length, branched; metanotum: medium length, branched, dense on posterior margin; S2–4 hair bands, long branched.

#### Description.

Male allotype: body length: 4.4 mm; headwidth: 1.6 mm. *Relative head measurements*: HW 50, ASD 2.4, AOD 6.6, HL 37, IAD 9.8, LFW 25, OAD 16, OOD 8.9, OW 17, UFW 33, HW/HL 1.4, LFW/UFW 0.8. *Relative wing measurements*: MSR 1.54, FSR 1.64, SFR 0.87.

*Structure*: terga anteriorly not depressed; BTP rounded. S7: dorsal apical lobe absent, ventral apical lobe large, branched setae present on apico medial area of ventral apical lobe.

*Sculpture*: scutum smooth with sparse punctures; metapostnotum shiny; T1 lineo-reticulate, T2–3 transverse lineo-reticulate; clypeus shiny, openly punctate; supraclypeal area shiny, openly to closely punctate; labrum; ocellocular area smooth, shiny, almost no punctures; frons smooth openly to sparsely punctate; vertex.

*coloration*: terga anteriorly brown-orange, posterior margins transparent white; mandibles brown at base, orange medially with brown tip; flagellum F1–3 black, F4–11 orange/brown below.

*Pubescence*: scutum: dispersed, medium length, branched; scutellum: dispersed, medium length, branched; metanotum: dispersed, medium length, branched; S2–4 with fringes of short hairs; scape shiny with medium length branched white hairs.

#### Remarks.

Four examined specimens have been DNA barcoded, accessible through the following links:

http://www.boldsystems.org/index.php/Public_RecordView?processid=AUSBS140-12

http://www.boldsystems.org/index.php/Public_RecordView?processid=AUSBS141-12

http://www.boldsystems.org/index.php/Public_RecordView?processid=AUSBS142-12

http://www.boldsystems.org/index.php/Public_RecordView?processid=AUSBS143-12

#### Flower records.

*Angianthusbrachypappus* (Asteraceae).

#### Distribution.

Figure 19F.

**Figure 19.**
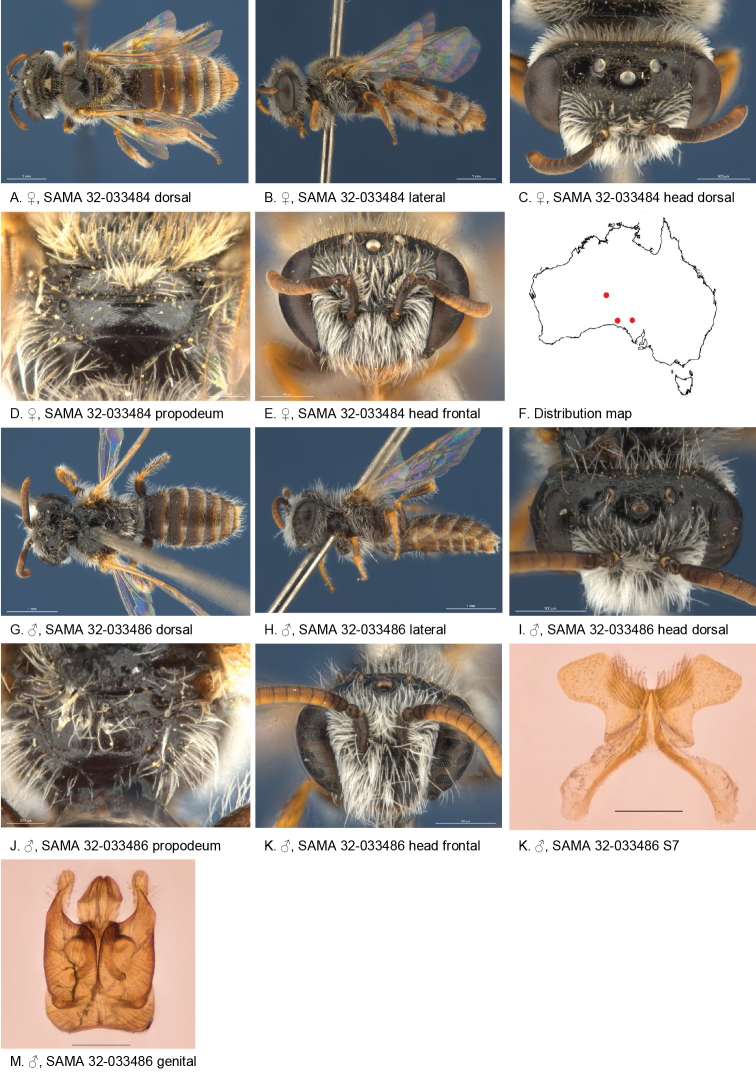
Leioproctus (Colletellus) laciniosus Leijs, sp. n. ♀ holotype and ♂ allotype. Scale bar: 0.1 mm (**K, M**).

#### Etymology.

The specific epithet refers to fringe of white hairs on the posterior T2–4.

### Leioproctus (Colletellus) longivultu

Taxon classificationAnimaliaHymenopteraColletidae

Leijs sp. n.

http://zoobank.org/317DB039-333A-4E08-88BA-9E69532873D5

Figure 20A–F

#### Specimens examined.

(4♀): Holotype female, 7 km E of Boologooro (24.2733S; 114.0308E), 27 Aug. 1980, Howard, C.A. & Houston, T.F., on *Calandriniapolyandra*, WAM 19841;

Paratypes 3 females, 10 km NNW of Meedo (25.6991S; 114.7175E), 23 Aug. 1980, Howard, C.A. & Houston, T.F., on *Calandriniapolyandra*, WAM 12373–4, 19838.

#### Diagnosis.

Ocellocular area smooth, sparse to openly punctate, metapostnotum dull, pit-reticulate, face longer that wide.

Male unknown.

#### Description.

Female: body length: 6.3 mm; head width: 2 mm. *Relative head measurements*: HW 50, ASD 3.1, AOD 8.5, HL 45, IAD 8.7, LFW 31, OAD 16, OOD 9.7, OW 17, UFW 34, HW/HL 1.1, LFW/UFW 0.9. *Relative wing measurements*: MSR 1.49, FSR 0.88, SFR 1.04.

*Structure*: terga anteriorly not depressed; BTP pointed; BTP/tibial length ratio 0.34; inner hind tibial spur pectinate with 3 large teeth; Clypeus ventral margin narrow, 1/4 of ocellar diameter.

*Sculpture*: scutum smooth, closely punctate; metapostnotum triangular shaped, dull, pit-reticulate; T1 lineo-reticulate, T2–3 transverse lineo-reticulate; clypeus smooth, closely punctate, supraclypeal area smooth, closely punctate; ocellocular area smooth openly punctate; frons smooth closely punctate; scape somewhat shiny.

*Coloration*: terga anteriorly brown-orange, posterior margins transparent orange; scopa light brown; labrum dark brown; mandibles orange with brown tip; brown; flagellum F4–10 orange brown.

*Pubescence*: scutum: very short, close, light brown; scutellum: very short, close, light brown; sterna 1–4 with fringes of long branched hairs, S5 with dense fringe of hairs.

#### Flower records.

*Calandriniapolyandra* (Montiaceae).

#### Distribution.

Figure 20F.

**Figure 20.**
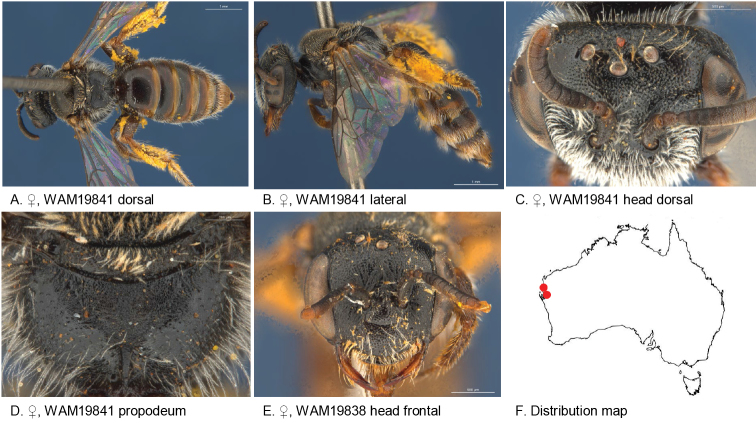
Leioproctus (Colletellus) longivultu Leijs, sp. n ♀ holotype.

#### Etymology.

The specific epithet refers to the elongated face of this species.

### Leioproctus (Colletellus) lucidus

Taxon classificationAnimaliaHymenopteraColletidae

Leijs sp. n.

http://zoobank.org/30B4E9A5-1DBC-4C6E-8DFA-7B72AD068E8F

Figure 21A–F

#### Specimens examined.

(1♀): Holotype female, 12 km ENE of Bungalbin Hill (30.29S; 119.69E), 11 Sep. 1979, Houston, T.F. et al., WAM 19833.

#### Diagnosis.

Integument of most body parts smooth and shiny, terga with narrow transparent posterior margins and open fringes of white hairs.

Male unknown.

#### Description.

Female: body length: 6 mm; head width: 1.9 mm. *Relative head measurements*: HW 50, ASD 3.2, AOD 8.0, HL 37, IAD 9.8, LFW 31, OAD 13, OOD 9.7, OW 17, UFW 34, HW/HL 1.4, LFW/UFW 0.9. *Relative wing measurements*: MSR 1.57, FSR 0.97, SFR 1.04.

*Structure*: terga anteriorly not depressed; BTP rounded; BTP/tibial length ratio 0.26; inner hind tibial spur pectinate with 5 strong teeth; clypeus ventral margin width about half ocellar diameter.

*Sculpture*: scutum smooth, openly punctate; metapostnotum smooth with shallow microsculpture; T1 lineo-reticulate, T2–3 transverse lineo-reticulate; clypeus smooth, openly to closely punctate; supraclypeal area smooth, openly to closely punctate; ocellocular area smooth, almost no punctures; frons smooth openly to closely punctate; scape below shiny with sparse punctures.

*Coloration*: terga anteriorly brown, posterior margins transparent orange; scopa light brown hairs; labrum dark-brown; mandibles red-brown darker at basis; scape dark-brown; flagellum dark brown, apical 7 segments brown.

*Pubescence*: scutum: medium to short, sparse brown; scutellum: medium to short, sparse brown; sterna S5 with fringe of dense white hairs.

#### Flower records.

No data.

#### Distribution.

Figure 21F.

**Figure 21.**
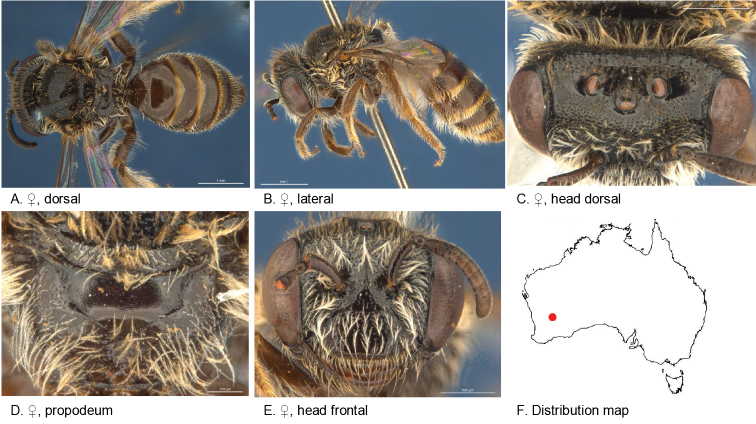
Leioproctus (Colletellus) lucidus Leijs, sp. n. ♀ holotype WAM19833.

#### Etymology.

The specific epithet refers to the smooth and shiny integument on head, scutum and terga of this species.

### Leioproctus (Colletellus) nitidifuscus

Taxon classificationAnimaliaHymenopteraColletidae

Leijs sp. n.

http://zoobank.org/1344DABE-B302-4945-8E61-B8C2B8553644

Figure 22A–F

#### Specimens examined.

(6♀): Holotype female, Kalbarri NP (27.8333S; 114.4667E), 14 Aug. 2003, Bickel, D., yellow pan trap, AM, K4473004.

Paratypes 5 females, same locality data as holotype, AM, K447300–03, 05.

#### Diagnosis.

Entire body smooth and shiny, abdomen brown, terga with transparent posterior margins.

Male unknown.

#### Description.

female holotype: body length: 4.8 mm; head width: 1.5 mm. *Relative head measurements*: HW 50, ASD 3.8, AOD 9.5, HL 43, IAD 9.9, LFW 34, OAD 15, OOD 10.5, OW 18, UFW 36, HW/HL 1.2, LFW/UFW 0.9. *Relative wing measurements*: MSR 1.92, FSR 1.16, SFR 1.09.

*Structure*: terga anteriorly not depressed; BTP rounded, elongated; BTP/tibial length ratio 0.28; inner hind tibial spur ciliate with circa 20 fine teeth.

*Sculpture*: scutum smooth, openly punctate without punctures on scutellum; metapostnotum almost entirely smooth, some transverse shallow reticulation medio-anteriorly, horizontal part 3 times as long as vertical; T1 lineo-reticulate, T2–3 transverse lineo-reticulate; clypeus smooth, sparse punctures; supraclypeal area smooth without punctures; labrum with row of orange setae; ocellocular area smooth, shiny, some scattered punctures near eye; frons smooth openly to sparsely punctate; scape dull, roughly sculptured with punctures and reticulation.

*Coloration*: terga anteriorly brown-orange, posterior margins transparent white; scopa white; mandibles brown, orange tip; flagellum dark, apical two segments with orange tint below.

*Pubescence*: scutum: open, short and some scattered, long; scutellum: almost bare; S2–4 with open medium length branched hairs, S5 with dense fringe of branched hairs.

#### Flower records.

No data.

#### Distribution.

Figure 22F.

**Figure 22.**
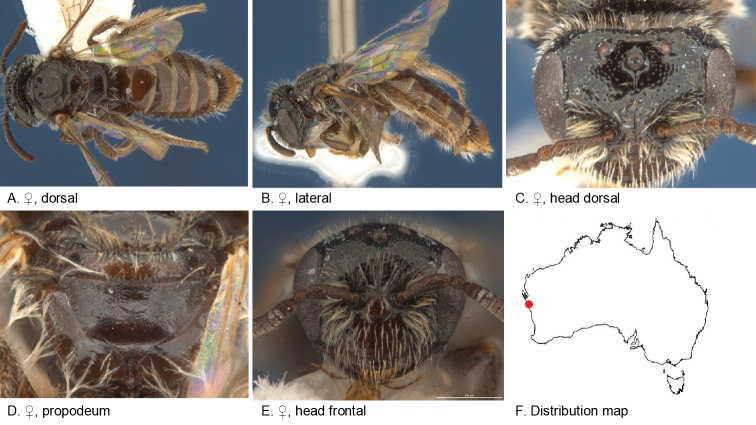
Leioproctus (Colletellus) nitidifuscus Leijs, sp. n. ♀ holotype AM K447304.

#### Etymology.

The specific epithet refers to very smooth and brown integument of large parts of the body of this species.

### Leioproctus (Colletellus) pectinatus

Taxon classificationAnimaliaHymenopteraColletidae

Leijs sp. n.

http://zoobank.org/F3DBB807-A671-4C61-BE15-9878922ED85C

Figure 23A–F

#### Specimens examined.

(4♀): Holotype female, 25 km SW of Tangadee (24.5688S; 118.7636E), 22 Aug. 1984, Houston, T.F. & Hanich, B.P., on *Calandrinia*, WAM 12333;

Paratypes female, 24 km NNE of Beyondie (24.7055S; 120.2558E), 17 Aug. 1984, Houston, T.F. & Hanich, B.P., on *Calandrinia*, WAM 12329; 2 females, 25 km SW of Tangadee (24.5688S; 118.7636E), 22 Aug. 1984, Houston, T.F. & Hanich, B.P., on *Calandrinia*, WAM 12331–2.

#### Diagnosis.

Ocellocular area openly punctate, propodeal triangle small, shiny and micro alveolate, inner hind tibial spur strongly pectinate.

Male unknown.

#### Description.

Female: body length: 6.7 mm; head width: 1.9 mm. *Relative head measurements*: HW 50, ASD 2.8, AOD 9.0, HL 44, IAD 10.0, LFW 32, OAD 17, OOD 9.3, OW 17, UFW 34, HW/HL 1.1, LFW/UFW 0.9. *Relative wing measurements*: MSR 1.38, FSR 1.00, SFR 0.93.

*Structure*: terga anteriorly little depressed; BTP rounded; BTP/tibial length ratio 0.18; inner hind tibial spur pectinate with 2–5 strong teeth; flagellum F1>F2, F1–9 wider than long.

*Sculpture*: scutum smooth, with close to dense punctation; metapostnotum shiny, finely microalveolate; T1 lineo-reticulate, T2–3 transverse lineo-reticulate; clypeus shiny, openly punctate, smooth between, anterior margin not drawn in thin plate; supraclypeal area shiny, openly punctate, smooth between; scape shiny; labrum medially slightly raised; ocellocular area punctate; frons punctate; vertex punctate.

*Coloration*: terga anteriorly black, posterior margins transparent orange, fovea on T2 black; scopa white; mandibles orange brown with darker tip; scape with white pubescence F1–9 black, orange towards the tip.

*Pubescence*: scutum: medium length, open; scutellum: medium long, open; metanotum medium length, open; sterna 1–4 with fringes of long branched hairs, S5 with dense fringe of hairs; scape with white pubescence.

#### Flower records.

*Calandrinia* sp. (Montiaceae).

#### Distribution.

Figure 23F.

**Figure 23.**
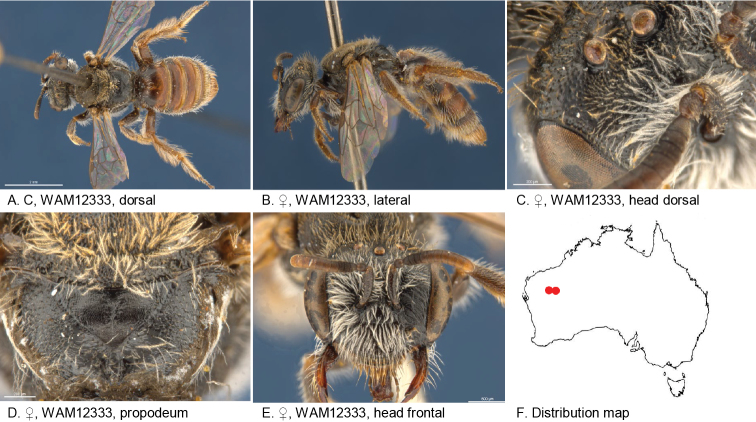
Leioproctus (Colletellus) pectinatus Leijs, sp. n. ♀ holotype.

#### Etymology.

The specific epithet refers to the strongly pectinate inner hind tibial spur.

### Leioproctus (Colletellus) pilotapilus

Taxon classificationAnimaliaHymenopteraColletidae

Leijs sp. n.

http://zoobank.org/CA4D2A0A-FD06-4220-A93B-22EAE0F4951F

Figure 24A–N

#### Specimens examined.

(3♂, 6♀): Holotype female, 41km E of Charlies Knob (24.6808S; 124.9858E), 01 Aug. 1983, Houston, T.F. & McMillan, R.P., on *Schoeniacassiniana*, WAM 12401;

Allotype male, WAM 12402 same data as holotype;

Paratypes 1 male , 41km E of Charlies Knob (24.6808S; 124.9858E), 01 Aug. 1983, Houston, T.F. & McMillan, R.P., on *Schoeniacassiniana*, WAM 12402-3; 1 female, 1 male, 11 km SW of Leake, Mount (25.8486S; 119.0769E), 16 Aug. 1984, Houston, T.F. & Hanich, B.P., on *Podolepiscanescens*, WAM 12404–5; male, Cohen, Lake (24.4508S; 125.0308E), 01 Aug. 1983, Houston, T.F. & McMillan, R.P., on *Schoeniacassiniana*, WAM 12406; female, Throssell, Lake (27.6230S; 124.1144E), 13 Sep. 1982, Houston, T.F. & Hanich, B.P., on *Helichrysum*?, WAM 12407; 2 females, 11 km NNE of Anketell (27.9872S; 118.9508E), 04 Sep. 1981, Houston, T.F., WAM 19836–7; female, 10 km NNW of Meedo (25.6991S; 114.7175E), 23 Aug. 1980, Howard, C.A. & Houston, T.F., on *Helipterumcraspedioides*, WAM 19840.

#### Diagnosis.

Ocellocular area smooth with dispersed punctures, metapostnotum with minute shallow pit-reticulation, female scutum covered with dense short brown pubescence, BTP rounded, inner hind tibial spur strongly pectinate, terga with transparent posterior margins.

#### Description.

Female holotype: body length: 6.6 mm; head width: 1.8 mm. *Relative head measurements*: HW 50, ASD 2.3, AOD 9.2, HL 39, IAD 9.4, LFW 31, OAD 16, OOD 9.9, OW 17, UFW 35, HW/HL 1.3, LFW/UFW 0.9. *Relative wing measurements*: MSR 1.22, FSR 1.00, SFR 0.86.

*Structure*: terga anteriorly little depressed; BTP rounded; BTP/tibial length ratio 0.16; inner hind tibial spur pectinate with 4 large spines.

*Sculpture*: scutum smooth, closely punctate; metapostnotum minute shallow pit-reticulate; T1 lineo-reticulate, T2–3 transverse lineo-reticulate; clypeus smooth, openly to closely punctate , ventral margin narrow brown; supraclypeal area smooth, openly to closely punctate; ocellocular area dull, finely pit-reticulate; frons smooth openly to closely punctate; scape shiny but not smooth.

*Coloration*: terga anteriorly brown, posterior margins wide, transparent orange; white lateral hair bands on T2–3, T4 entire; scopa white; mandibles brown with dark tip; labrum brown; scape black; flagellum brown-black.

*Pubescence*: scutum: short, close, brown; scutellum: short, close, brown; S2–4 with rows of long openly spaced branched hairs, S5 with fringe of closely spaced branched hairs.

#### Description.

Male: measurements: body length: 5.9 mm; head width: 1.85 mm. *Relative head measurements*: HW 50, ASD 2.8, AOD 7.3, HL 41, IAD 8.7, LFW 28, OAD 15, OOD 10.3, OW 18, UFW 36, HW/HL 1.2, LFW/UFW 0.8. *Relative wing measurements*: MSR 1.24, FSR 0.93, SFR 1.00.

*Structure*: terga anteriorly little depressed; BTP rounded; F4–11 about as wide as long. S7: dorsal apical lobe absent, ventral apical lobe large, with robust simple setae on ventral apical lobe.

*Sculpture*: scutum smooth with microsculpture, sparsely punctate; metapostnotum dullish, minute shallow pit-reticulate, some transverse striation medio-anteriorly; T1 lineo-reticulate, T2–3 transverse lineo-reticulate; clypeus smooth, openly to closely punctate ; supraclypeal area smooth, openly to closely punctate ; ocellocular area shiny, micro-reticulate; frons smooth openly to closely punctate; scape dull.

*Coloration*: terga anteriorly brown-black, posterior margins wide, transparent orange; mandibles brown with reddish tip; labrum black; scape brown-black; flagellum brown.

*Pubescence*: scutum: long, open, whitish; scutellum: long, open, whitish; scape with white, plumose pubescence.

#### Flower records.

*Helipterumcraspedioides* (Asteraceae), *Helichrysum*? sp. (Asteraceae), *Podolepiscanescens* (Asteraceae), *Schoeniacassiniana* (Asteraceae).

#### Distribution.

Figure 24F.

**Figure 24.**
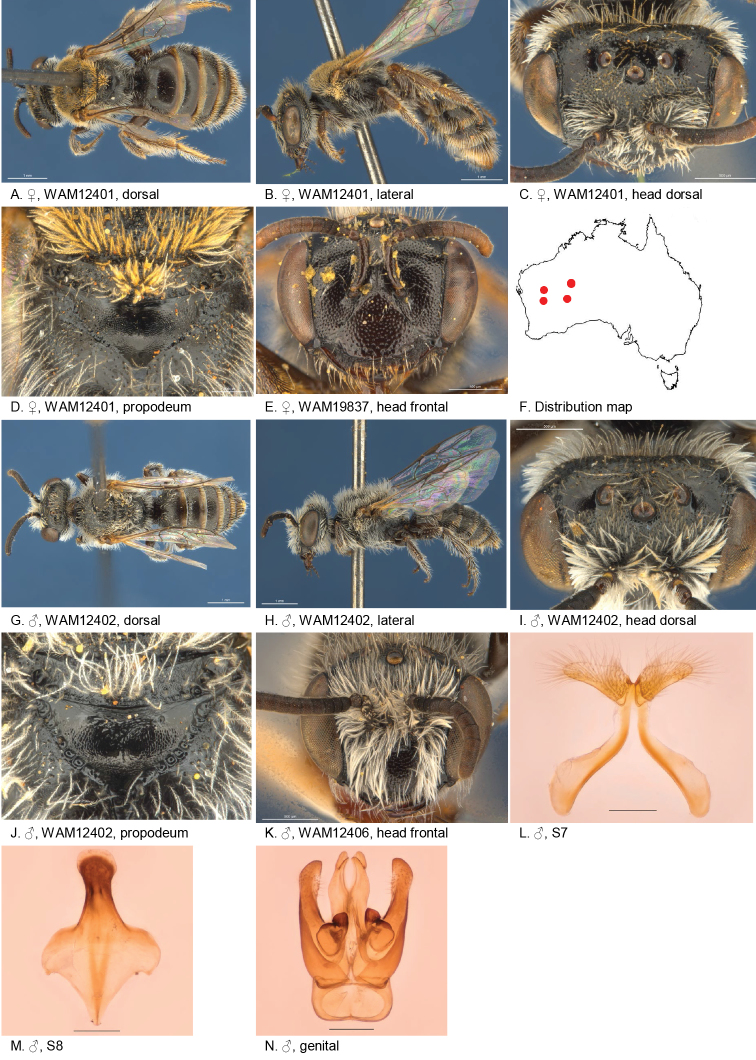
Leioproctus (Colletellus) pilotapilus Leijs, sp. n. ♀ holotype and ♂ allotype. Scale bar: 0.1 mm (**L, M, N**).

#### Etymology.

The specific epithet refers to the dense and short pubescence on the scutum.

### Leioproctus (Colletellus) quadripinnatus

Taxon classificationAnimaliaHymenopteraColletidae

Leijs sp. n.

http://zoobank.org/6FC7C6B3-9D13-4123-8750-ACEFC0B557D7

Figure 25A–I

#### Specimens examined.

(1♂): Holotype male, Eagle Bay (33.5591S; 115.0692E), 27 Sep. 1975, Spencer, K., WAM 19115.

#### Diagnosis.

Ocellocular area smooth with a few sparse fine punctures, metapostnotum entirely smooth, S7 slender dorsal and ventral apical lobes bearing robust simple setae.

Female unknown.

#### Description.

Male holotype: body length: 4.5 mm; head width: 1.5 mm. *Relative head measurements*: HW 50, ASD 3.3, AOD 5.9, HL 45, IAD 11.1, LFW 27, OAD 15, OOD 12.2, OW 18, UFW 37, HW/HL 1.1, LFW/UFW 0.7. *Relative wing measurements*: MSR 1.43, FSR 0.91, SFR 1.19.

*Structure*: terga anteriorly little depressed; BTP rounded, very short; flagellum short, F1–9 wider than long. S7: dorsal apical lobe slender, ventral apical lobe slender, robust simple setae present on dorsal subcentral apical ridge and ventral apical lobe; scape short.

*Sculpture*: scutum smooth sparsely punctate; metapostnotum smooth, without or very shallow microsculpture, almost wholly vertical; T1 lineo-reticulate, T2–3 transverse lineo-reticulate; clypeus shiny, openly punctate; supraclypeal area shiny, openly punctate; labrum orange; ocellocular area smooth with sparse fine punctures; scape dull.

*Coloration*: terga anteriorly brown, posterior margins transparent pale orange; mandibles brown with lighter tip.

*Pubescence*: scutum: medium length sparse, light brown; scutellum: medium length, sparse, light brown.

#### Flower records.

No data.

#### Distribution.

Figure 25I.

**Figure 25.**
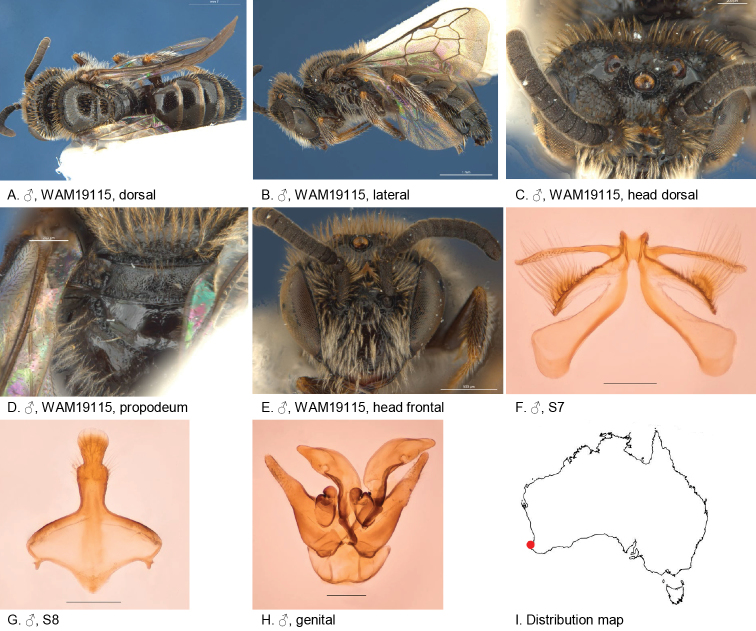
Leioproctus (Colletellus) quadripinnatus Leijs, sp. n. ♂ holotype. Scale bar: 0.1 mm (**F, G, H**).

#### Etymology.

The specific epithet refers to thin feathery like apical lobes of the male sterna 7.

### Leioproctus (Colletellus) rubicundus

Taxon classificationAnimaliaHymenopteraColletidae

Leijs sp. n.

http://zoobank.org/A33ADBE8-8CF0-487B-9D54-F79F69DA6F42

Figures 2A , 26A–N

#### Specimens examined.

(2♂, 5♀): Holotype female, Bon Bon Stn (30.7789S; 135.3841E), 26 Oct. 2010, Leijs, R., on *Angianthusbrachypappus*, SAMA 32-033488 AUSBS145-12/RL1630A;

Allotype male, Pernatty Stn, North Tiffin Hill (31.4826S; 137.7454E), 05 Sep. 2016, Leijs, R., on *Gunniopsis*, SAMA 32-033492, KR4734;

Paratypes 2 females, SAMA 32-033487, 89; BOLD: AUSBS139-12/RL1631; AUSBS144-12/RL1631B same locality data as holotype; 2 females, 1 male, Pernatty Stn, North Tiffin Hill (31.4826S; 137.7454E), 05 Sep. 2016, Leijs, R., on *Gunniopsis*, SAMA 32-033490,91,93 KR4732,33,35; 1 female, from salt lake, SA, (30.8161S; 134.3042E), P. Hudson.

#### Diagnosis.

First recurrent vein meeting to the first submarginal cross vein, ocellocular area smooth, without punctures, metapostnotum smooth, shiny, horizontal part shorter than vertical, T2–4 with semi erect hairs on posterior margins, female BTB rounded, inner hind tibial spur pectinate.

#### Description.

Female holotype: body length: 6.1 mm; head width: 2.1 mm. *Relative head measurements*: HW 50, ASD 3.0, AOD 8.9, HL 34, IAD 8.6, LFW 33, OAD 15, OOD 9.0, OW 18, UFW 35, HW/HL 1.5, LFW/UFW 0.9. *Relative wing measurements*: MSR 1.63, FSR 1.72, SFR 0.84.

*Structure*: terga anteriorly not depressed; BTP rounded; BTP/tibial length ratio 0.21; inner hind tibial spur pectinate with 10 teeth.

*Sculpture*: scutum smooth with sparse punctures; metapostnotum smooth, shiny, horizontal part shorter than vertical; T1 lineo-reticulate, T2–3 transverse lineo-reticulate; clypeus shiny, closely punctate, orange anterior rim; supraclypeal area shiny, closely punctate; ocellocular area smooth, shiny; frons smooth openly to sparsely punctate; scape shiny, sparsely punctate.

*Coloration*: terga anteriorly orange, laterally brown medially, posteriorly orange, transparent posterior margins, no adpressed hair bands; scopa white; mandibles orange with brown tip; flagellum F1–3 black, F4–10 orange/brown below; scape black.

*Pubescence*: scutum: dispersed, medium length, branched; scutellum: dispersed, medium length, branched; metanotum: dispersed, medium length, branched; S2–4 hair bands, long, branched.

#### Description.

Male allotype: body length: 5.5 mm; head width: 1.7 mm. *Relative head measurements*: HW 50, ASD 2.5, AOD 7.9, HL 38, IAD 9.4, LFW 27, OAD 15, OOD 8.6, OW 17, UFW 33, HW/HL 1.3, LFW/UFW 0.8. *Relative wing measurements*: MSR 1.72, FSR 1.39, SFR 1.00.

*Structure*: terga anteriorly not depressed; BTP rounded; flagellum F1–3 black, F 4–11 brown below, shorter than wide.

*Sculpture*: scutum smooth with almost no punctures; metapostnotum shiny with microsculpture, horizontal part shorter than vertical; T1 lineo-reticulate, T2–3 transverse lineo-reticulate; clypeus shiny, closely punctate; supraclypeal area shiny, closely punctate; ocellocular area smooth, shiny, almost no punctures; frons smooth, openly to closely punctate; scape shiny, sparsely punctate.

*Coloration*: terga anteriorly black, posterior margins transparent orange; mandibles black with brown tip; scape black.

*Pubescence*: scutum: white, dispersed, medium length, branched; scutellum: white, dispersed, medium length, branched; S2–4 short dense apical hair bands.

S7: dorsal apical lobe absent, ventral apical lobe large, branched setae present on apico-medial area of ventral apical lobe.

#### Remarks.

Three specimens have been DNA barcoded, accessible through the following links:

http://www.boldsystems.org/index.php/Public_RecordView?processid=AUSBS139-12

http://www.boldsystems.org/index.php/Public_RecordView?processid=AUSBS144-12

http://www.boldsystems.org/index.php/Public_RecordView?processid=AUSBS145-12

#### Flower records.

*Angianthusbrachypappus* (Asteraceae), *Gunniopsis* sp. (Aizoaceae).

#### Distribution.

Figure 26F.

**Figure 26.**
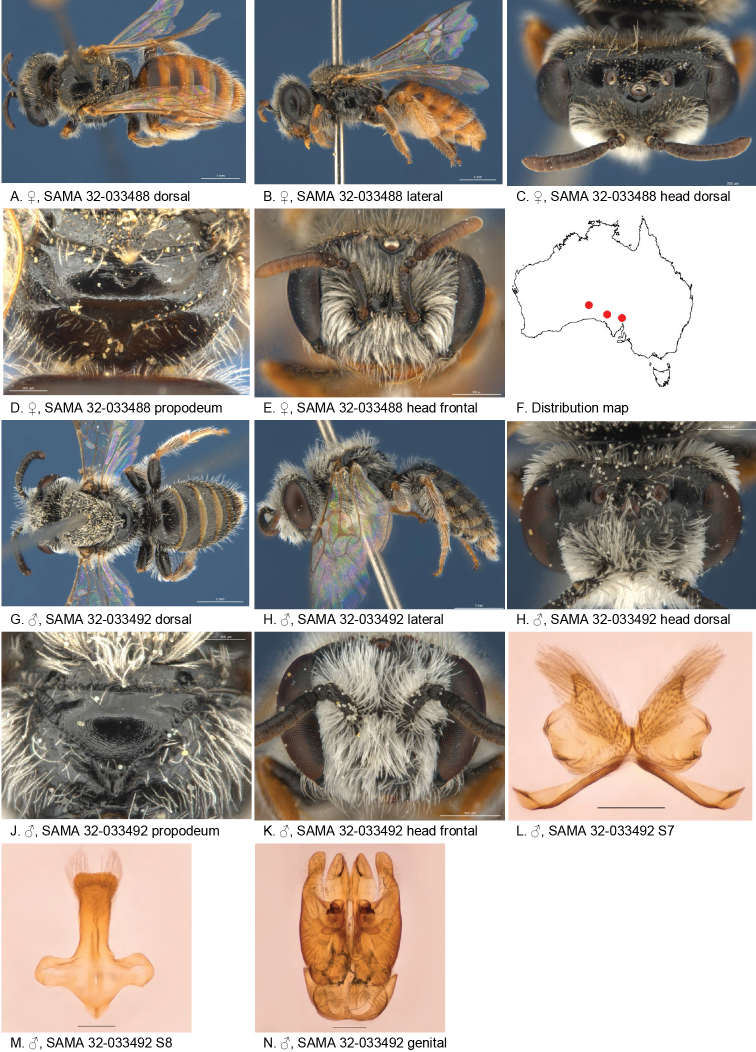
Leioproctus (Colletellus) rubicundus Leijs, sp. n. ♀ holotype and ♂ allotype. Scale bar: 0.1 mm (**L, M, N**).

#### Etymology.

The specific epithet refers to the colour of the female metasoma.

### Leioproctus (Colletellus) rubricinctus

Taxon classificationAnimaliaHymenopteraColletidae

Leijs sp. n.

http://zoobank.org/A754B7A1-FEC3-4602-98A4-1A03EC0FC877

Figure 27A–H

#### Specimens examined.

(10♂): Holotype male, Bullfinch (30.5797S; 119.1144E), 07 Sep. 1979, Houston, T.F. et al., WAM 12357;

Paratypes 5 males, WAM 12356,58–61 same locality data as holotype; 3 males, Bullfinch (30.7147S; 119.1144E), 07 Sep. 1979, Houston, T.F. et al., on *Thryptomenetuberculata*, WAM 12362–4; 1 male, Kadji Kadji (29.1833S; 116.458E), 19 Sep. 2009, Charles Darwin Bush Blitz, Leijs, R., blue pan trap, SAMA 32-033499/RL1526.

#### Diagnosis.

Ocellocular area dull, densely punctate and irregular roughened, frons irregular pit-reticulate, terga with pregradular depressions, posterior margins broadly orange transparent, F3–9 longer than wide.

Female unknown.

#### Description.

Male holotype: body length: 4.9 mm; head width: 1.7 mm. *Relative head measurements*: HW 50, ASD 3.2, AOD 6.0, HL 37, IAD 11.5, LFW 26, OAD 14, OOD 10.9, OW 16, UFW 36, HW/HL 1.3, LFW/UFW 0.7. *Relative wing measurements*: MSR 1.56, FSR 1.00, SFR 1.04.

*Structure*: terga anteriorly depressed; BTP rounded; flagellum F1=F2, long, F3–9 longer than wide. S7: dorsal apical lobe absent, ventral apical lobe large, branched setae present on posterior area of dorsal subcentral ridge.

*Sculpture*: scutum dull, transverse lineo-reticulate and openly punctate; metapostnotum pit-reticulate; T1 lineo-reticulate, T2–3 transverse lineo-reticulate; clypeus dull, finely irregularly roughened; supraclypeal area somewhat shiny, finely irregularly roughened; scape short, dull; ocellocular area dull, densely punctate and irregularly roughened; frons irregular pit-reticulate.

*Coloration*: terga anteriorly orange, posterior margins transparent orange; labrum orange; mandibles black with red-brown tip.

*Pubescence*: scutum: short, fine and dispersed, long, off-white; scutellum: short, fine, with dispersed, long off-white; scape with long white pubescence.

#### Flower records.

*Thryptomenetuberculate* (Myrtacea).

#### Distribution.

Figure 27H.

**Figure 27.**
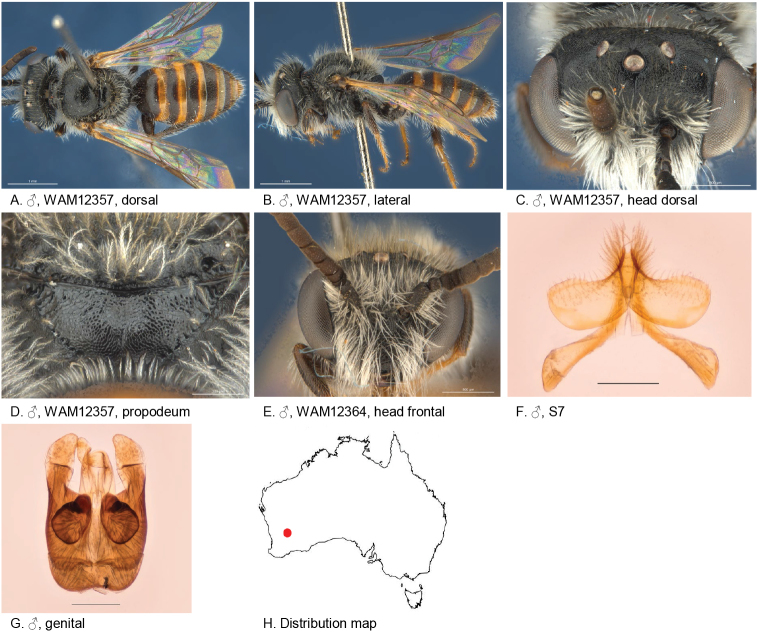
Leioproctus (Colletellus) rubricinctus Leijs, sp. n. ♂ holotype. Scale bar: 0.1 mm (**F, G**).

#### Etymology.

The specific epithet refers to the red colouration of the anterior parts of the T2–4.

### Leioproctus (Colletellus) similis

Taxon classificationAnimaliaHymenopteraColletidae

Leijs sp. n.

http://zoobank.org/A801B9A1-9DF9-4CE7-A2E8-E4227C8358FF

Figure 28A–F

#### Specimens examined.

(4♀): Holotype female, Mt Gibson Stn (29.8014S; 117.4028E), 28 Aug. 2001, Leijs, R., on *Borya*, SAMA 32-033482;

Paratypes 2 females, Paynes Find (29.2636S; 117.6831E), 01 Aug. 1982, Main, B.Y., WAM 12366–7; female, 22 km E of Bullfinch (30.7866S; 119.1144E), 18 Sep. 1979, Houston, T.F. et al., WAM 12380.

#### Diagnosis.

Ocellocular area dull, finely roughened, frons dull roughly pit-reticulate, scutum medium length dense brown pubescence, terga dark with brown transparent posterior margins.

Male unknown.

#### Description.

Female holotype: body length: 5.8 mm; head width: 2 mm. *Relative head measurements*: HW 50, ASD 2.8, AOD 8.4, HL 37, IAD 11.7, LFW 31, OAD 12, OOD 9.5, OW 17, UFW 35, HW/HL 1.4, LFW/UFW 0.9. *Relative wing measurements*: MSR 1.68, FSR 1.00, SFR 1.10.

*Structure*: terga anteriorly; BTP pointed; BTP/tibial length ratio 0.3; inner hind tibial spur ciliate.

*Sculpture*: scutum anteriorly transverse lineo-reticulate, densely punctate; metapostnotum fine pit-reticulate; T1 lineo-reticulate, T2–3 transverse lineo-reticulate; clypeus dull, microroughened, with open large punctures; supraclypeal area somewhat shiny, with microsculpture without punctures; ocellocular area dull, finely roughened; frons dull roughly pit-reticulate.

*Coloration*: terga anteriorly brown-orange, posterior margins transparent orange; scopa brown; labrum black; mandibles black; scape black; flagellum black.

*Pubescence*: scutum: medium length, brown; scutellum: medium length, brown; S2–4 medially on posterior margin with long simple hairs.

#### Flower records.

*Borya* sp. (Boryaceae).

#### Distribution.

Figure 28F.

**Figure 28.**
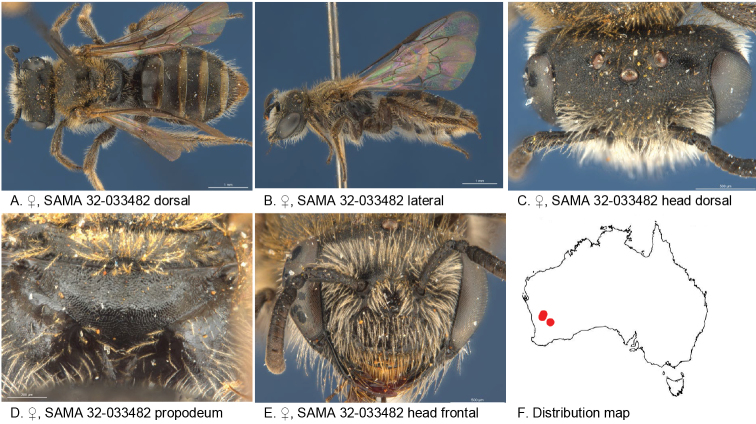
Leioproctus (Colletellus) similis Leijs, sp. n. ♀ holotype.

#### Etymology.

The specific epithet refers to the common habitus of this species within this subgenus.

### Leioproctus (Colletellus) splendens

Taxon classificationAnimaliaHymenopteraColletidae

Leijs sp. n.

http://zoobank.org/9995F3B5-0D0E-407D-BD8E-89C2EC665356

Figure 29A–F

#### Specimens examined.

(2♀): Holotype female, 14 km SE of Tamala (26.3666S; 113.9817E), 28 Aug. 1997, Houston, T.F. & Mathiasen, P., on *Calandrinia*, WAM 21484;

Paratype female, same locality data as holotype, WAM 21485.

#### Diagnosis.

Integument of metapostnotum and terga smooth and shiny without punctures, ocellocular area smooth, closely punctured.

Male unknown.

#### Description.

Female holotype: body length: 4.5 mm; head width: 1.5 mm. *Relative head measurements*: HW 50, ASD 2.7, AOD 7.7, HL 40, IAD 11.5, LFW 31, OAD 16, OOD 9.3, OW 17, UFW 34, HW/HL 1.3, LFW/UFW 0.9. *Relative wing measurements*: MSR 1.68, FSR 1.21, SFR 1.13.

*Structure*: terga anteriorly little depressed; BTP pointed; BTP/tibial length ratio 0.23; inner hind tibial spur ciliate with circa 18 small teeth.

*Sculpture*: scutum smooth, closely punctate; metapostnotum minute shallow pit-reticulate; T1 lineo-reticulate, T2–3 transverse lineo-reticulate; clypeus smooth with microsctructure, openly punctate, ventral margin broad; supraclypeal area smooth with microsculpture, sparsely punctate; ocellocular area smooth, closely punctured; frons densely punctate; scape dull.

*Coloration*: terga anteriorly brown-orange, posterior margins wide, transparent orange; scopa light brown; labrum black; mandibles black with brown tip; scape brown-black; flagellum dark brown, last 5 segments light brown below.

*Pubescence*: scutum: short, close, brown; scutellum: short, close, brown.

#### Flower records.

*Calandrinia* sp. (Montiaceae).

#### Distribution.

Figure 29F.

**Figure 29.**
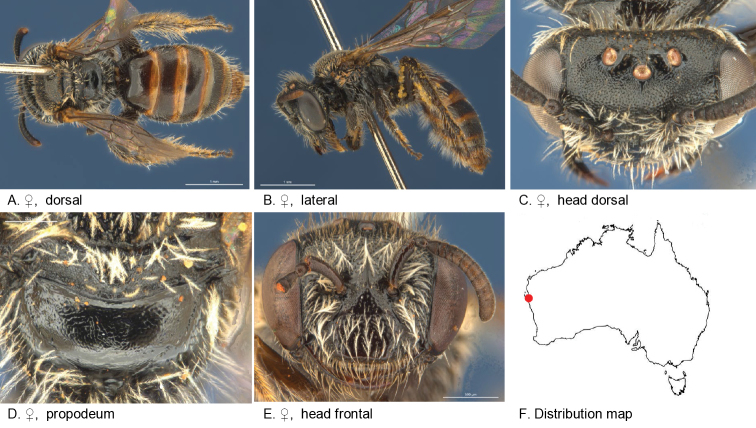
Leioproctus (Colletellus) splendens Leijs, sp. n. ♀ holotype WAM21484.

#### Etymology.

The specific epithet refers to the smooth and shiny integument on metapostnotum and terga of this species.

### Leioproctus (Colletellus) submetallicus

Taxon classificationAnimaliaHymenopteraColletidae

Leijs sp. n.

http://zoobank.org/0E3D2719-C8C5-4D19-9145-5D10E8A03A6E

Figure 30A–N

#### Specimens examined.

(2♀, 1♂): Holotype female, Mt Gibson Stn (29.6870S; 117.3723E), 23 Aug. 2001, Leijs, R., on *Grevillea*, SAMA 32-033496;

Allotype male, Northampton (28.08S; 114.67E), 11 July 1959, ANIC 32-111877;

Paratype female, Mt Gibson Homestead (29.6075S; 117.4108E), 29 Aug. 2001, Leijs, R., SAMA 32-033497.

#### Diagnosis.

The only species with faint metallic integument of head and thorax.

#### Description.

Female holotype: body length: 6 mm; head width: 1.9 mm. *Relative head measurements*: HW 50, ASD 3.3, AOD 8.4, HL 38, IAD 10.0, LFW 29, OAD 13, OOD 11.3, OW 16, UFW 36, HW/HL 1.3, LFW/UFW 0.8. *Relative wing measurements*: MSR 1.61, FSR 1.18, SFR 1.00.

*Structure*: terga anteriorly not depressed; BTP pointy; BTP/tibial length ratio 0.26; inner hind tibial spur ciliate; flagellum short, F1–9 wider than long.

*Sculpture*: scutum dull, transverse lineo-reticulate and openly punctate, with faint metallic shine; metapostnotum transverse lineo-reticulate to finely irregular roughened; T1 lineo-reticulate, T2–3 transverse lineo-reticulate; clypeus large openly punctate, microsculpture between punctures; supraclypeal area small densely punctate; scape relatively slender about 5 times as long as wide proximally; ocellocular area minutely roughened; frons irregularly pit-reticulate with faint metallic shine.

*Coloration*: terga anteriorly brown, posterior margins almost not transparent; scopa light brown, lighter ventrally; labrum black; mandibles black with brown tip.

*Pubescence*: scutum: short, orange-brown, with sparse long hairs; scutellum: short, orange-brown, with sparse long hairs; S5 with dense fringe of white hairs.

#### Description.

Male: body length: 5.9 mm; head width: 1.85 mm. *Relative head measurements*: HW 50, ASD 3.4, AOD 6.3, HL 40, IAD 9.2, LFW 24, OAD 13.8, OOD 10.9, OW 16.7, UFW 36.2, HW/HL 1.3, LFW/UFW 0.67. *Relative wing measurement*: MSR 1.26, FSR 1.24, SFR 1.0.

*Structure*: terga anteriorly almost not depressed; BTP short, almost not pointy; flagellum F1 < F2, F1–10 wider than long, F11 longer than wide. S7: dorsal apical lobe absent, ventral apical lobe large, branched setae present on dorsal subcentral apical ridge.

*Sculpture*: scutum dull, transverse lineo-reticulate and openly punctate, with faint metallic shine; metapostnotum transverse lineo-reticulate to finely irregular roughened; T1–3 lineo-reticulate; clypeus large openly to closely punctate, rough microsculpture between punctures; supraclypeal area small densely punctate; scape almost 4 times longer than wide, roughly sculptured; ocellocular area minutely roughened; frons irregularly pit-reticulate.

*Coloration* terga anteriorly black, posterior margins almost not transparent, brown; mandibles black with brown tip.

*Pubescence*: scutum and scutellum: open, long, branched, light brown; face with long white branched pubescence.

#### Flower records.

*Grevillea* sp. (Proteaceae).

#### Distribution.

Figure 30F.

**Figure 30.**
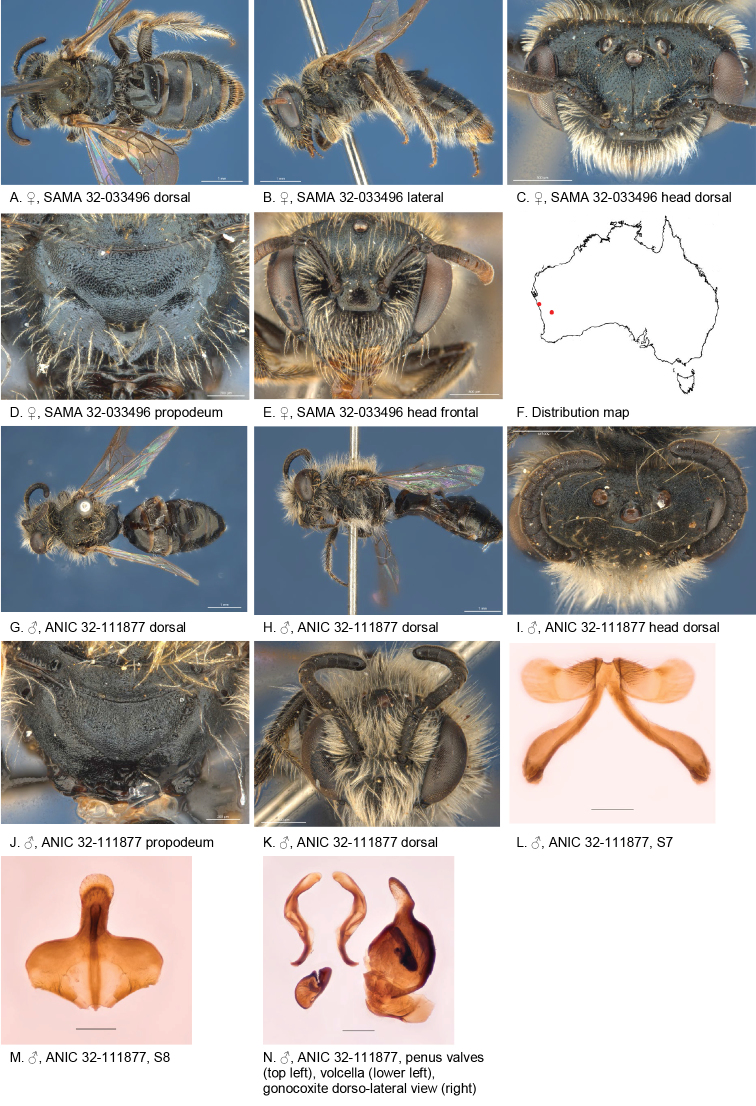
Leioproctus (Colletellus) submetallicus Leijs, sp. n. ♀ holotype and ♂ allotype.

#### Etymology.

The specific epithet refers to the faint metallic shine on the head and thorax.

### Leioproctus (Colletellus) velutinellus

Taxon classificationAnimaliaHymenopteraColletidae

Michener, 1965

Figure 31A–N

#### Specimens examined.

(24♂, 7♀): Holotype female, Kojarena nr. Geraldton, West Australia, 6 Sep. 1926, Nicholdson, K95546, AM;

Allotype male, Gooseberry Hill (31.9541S; 116.0469E), 07 Oct. 1994, Houston, T.F., on *Stylidium*, WAM 14417;

Other specimens: 5 males, Gooseberry Hill (31.9541S; 116.0469E), 09 Oct. 1986, Houston, T.F., on *Stylidiumbulbiferum*, WAM 12412–6; 3 males, 1 female, Gooseberry Hill (31.9541S; 116.0469E), 07 Oct. 1994, Houston, T.F., on *Stylidium*, WAM 14411,14–16; male, Gooseberry Hill (31.9541S; 116.0469E), 07 Oct. 1994, Houston, T.F., WAM 14418; female, Gooseberry Hill (31.9550S; 116.0489E), 15 Oct. 2009, Batley, M., on *Stylidiumbulbiferum*, AM 359774; 3 females, 63 km NW of Perth (32.3536S; 116.3306E), 21 Nov. 1981, Howard, C.A. & Houston, T.F., on Stylidiumneardivaricatum & *bulbiferum*, WAM 12417–9; male, Glen Forrest (31.9111S; 116.0992E), 19 Sep. 1976, Postmus, S.M., WAM 12420; male, Garden Island (32.205S; 115.6733E), 12 Nov. 1975, Postmus, S.M., WAM 14419; female, Walyunga National Park (31.7213S; 116.0708E), 17 Oct. 1993, Houston, T.F., on *Boronia* , WAM 14420; male, Burma Road Nature Reserve (29.0000S; 115.0831E), 15 Sep. 1986, McMillan, R.P., WAM 22322; 11 males, Lesmurdie (32.0125S; 116.0328E), 14 Oct. 2009, Batley, M., on *Stylidiumbulbiferum*, AM 359756–63,66–67, 361121.

#### Diagnosis.

Ocellocular area dull, minutely and very densely roughened, frons dull, minutely roughened, vertex dull, minutely roughened, metapostnotum slightly striate in lateral corners, terga with post gradular depressions, scutum covered with dense short brown pubescence.

#### Description.

Female WAM14415: body length: 5.9 mm; head width: 2 mm. *Relative head measurements*: HW 50, ASD 2.9, AOD 8.0, HL 35, IAD 10.0, LFW 29, OAD 13, OOD 10.6, OW 15, UFW 34, HW/HL 1.4, LFW/UFW 0.9. *Relative wing measurements*: MSR 1.33, FSR 1.08, SFR 0.93.

*Structure*: terga anteriorly depressed; BTP pointed, long; BTP/tibial length ratio 0.35; inner hind tibial spur ciliate with circa 22 little teeth.

*Sculpture*: scutum dull, densely punctate; metapostnotum shiny, with dense contiguous depressions, minutely striate in lateral corners; scape dull; T1 lineo-reticulate, T2–3 transverse lineo-reticulate; clypeus shiny, openly punctate, with micro depressions between punctures, ventral margin drawn into thin plate; supraclypeal area without punctures, only micro depressions; labrum smooth; ocellocular area dull, very dense micro roughened; frons dull, micro roughened; vertex dull, micro-roughened.

*Coloration*: terga anteriorly orange, posterior margins transparent orange; scopa white; labrum black; mandibles black with brown tip; flagellum F1–3 black, F4–10 little lighter below.

*Pubescence*: scutum: light brown, dense, short, branched; scutellum: dense, short, branched; metanotum: dense, short, branched; S1–4 with fringes of long branched hairs, S5 with dense fringe of hairs; scape with open medium length blond pubescence.

#### Description.

Male: body length: 5.4 mm; head width: 1.9 mm. *Relative head measurements*: HW 50, ASD 2.6, AOD 6.9, HL 36, IAD 11.4, LFW 26, OAD 12, OOD 10.0, OW 18, UFW 36, HW/HL 1.4, LFW/UFW 0.7. *Relative wing measurement*: MSR 1.39.

*Structure*: terga anteriorly depressed; BTP pointed; flagellum F1 > F2, F1–3 wider than long, F4–11 slightly longer than wide. S7: dorsal apical lobe absent, ventral apical lobe large, branched setae present on dorsal subcentral apical ridge.

*Sculpture*: scutum dull, densely punctate; metapostnotum shiny, with dense contiguous depressions, slightly striate in lateral corners; scape short, dull; T1 lineo-reticulate, T2–3 transverse lineo-reticulate; clypeus dense fine punctate, large punctures only at margins; supraclypeal area openly to sparsely punctate with underlying dense fine punctures; ocellocular area dull, very dense micro-roughened; frons dull, micro-roughened.

*Coloration*: terga anteriorly black-brown; terga posterior margins transparent orange; mandibles black with brown tip.

*Pubescence*: scutum: dense, short, branched; scutellum: dense, short, branched; metanotum: dense, short, branched; scape with long pubescence.

#### Remarks.

The female of this species is redescribed based on specimen WAM 14415, however comparison with the holotype did not reveal differences. The male of the species is described here for the first time. While Michener (2007) mentioned that the male genitalia and hidden sterna were illustrated by Michener (1965), these images could not be found in the referred publication, which explicitly states that the subgenus is only known in the female.

#### Flower records.

*Boronia* (Rutaceae), *Stylidiumbulbiferum* (Stylidiaceae), Stylidiumnrdivaricatum (Stylidiaceae), *Stylidium* sp. (Stylidiaceae).

#### Distribution.

Figure 31N.

**Figure 31.**
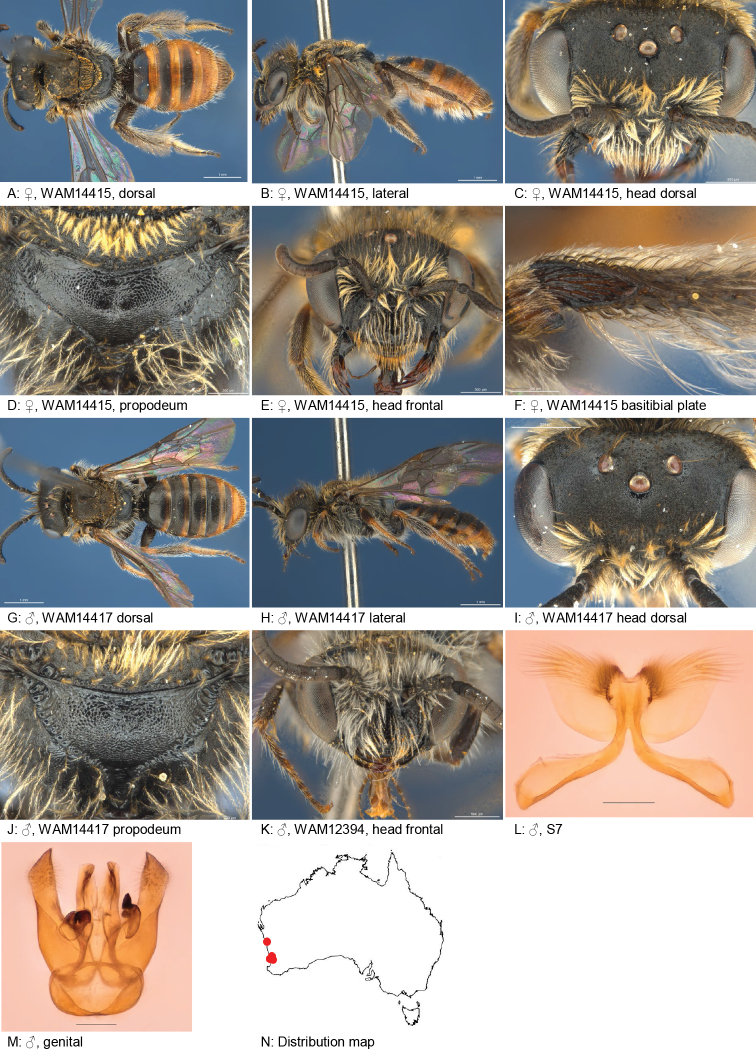
Leioproctus (Colletellus) velutinellus Michener, ♀ WAM14415, ♂ allotype. Scale bar: 0.1 mm (**L, M, N**).

## Supplementary Material

XML Treatment for Leioproctus Subgenus Colletellus

XML Treatment for Leioproctus (Colletellus) aberrans

XML Treatment for Leioproctus (Colletellus) alatus

XML Treatment for Leioproctus (Colletellus) albipilosus

XML Treatment for Leioproctus (Colletellus) albiscopis

XML Treatment for Leioproctus (Colletellus) aliceafontanus

XML Treatment for Leioproctus (Colletellus) altispinosus

XML Treatment for Leioproctus (Colletellus) aratus

XML Treatment for Leioproctus (Colletellus) auricorneus

XML Treatment for Leioproctus (Colletellus) bidentatus

XML Treatment for Leioproctus (Colletellus) centralis

XML Treatment for Leioproctus (Colletellus) ciliatus

XML Treatment for Leioproctus (Colletellus) claviger

XML Treatment for Leioproctus (Colletellus) consobrinus

XML Treatment for Leioproctus (Colletellus) constrictus

XML Treatment for Leioproctus (Colletellus) laciniosus

XML Treatment for Leioproctus (Colletellus) longivultu

XML Treatment for Leioproctus (Colletellus) lucidus

XML Treatment for Leioproctus (Colletellus) nitidifuscus

XML Treatment for Leioproctus (Colletellus) pectinatus

XML Treatment for Leioproctus (Colletellus) pilotapilus

XML Treatment for Leioproctus (Colletellus) quadripinnatus

XML Treatment for Leioproctus (Colletellus) rubicundus

XML Treatment for Leioproctus (Colletellus) rubricinctus

XML Treatment for Leioproctus (Colletellus) similis

XML Treatment for Leioproctus (Colletellus) splendens

XML Treatment for Leioproctus (Colletellus) submetallicus

XML Treatment for Leioproctus (Colletellus) velutinellus

## Supplementary materials

Supplementary material 1 Supplementary table – specimen info Data type: Microsoft Excel Worksheet (.xlsx) Explanation note: species data This is an open access article distributed under the terms of the Creative Commons Attribution License (CC BY 4.0), which permits unrestricted use, distribution, and reproduction in any medium, provided the original author and source are credited. Remko Leijs, James Dorey, Katja Hogendoorn
